# Can artificial intelligence improve precision dosing? a systematic review of narrow therapeutic index drugs

**DOI:** 10.1186/s12911-026-03769-7

**Published:** 2026-08-17

**Authors:** Ahmed Alaqab, Mohammed Alfaqeeh, Sofa D. Alfian, Sidik Maulana, Safwan Mahmood AL-selwi, Anas Hamad, Jan-Willem Alffenaar, Abdullah A. H. Othman

**Affiliations:** 1 Department of Pharmacy, Faculty of Medical Sciences, Al-Ruwad University, Taiz, Republic of Yemen; 2 Department of Pharmacology and Clinical Pharmacy, Faculty of Pharmacy, Universitas Padjadjaran, Bandung, Indonesia; 3 Centre of Excellence for Pharmaceutical Care Innovation, Universitas Padjadjaran, Bandung, Indonesia; 4 Center for Health Technology Assessment, Universitas Padjadjaran, Bandung, Indonesia; 5 Doctoral Program of Nursing, Faculty of Nursing, Universitas Padjadjaran, Bandung, Indonesia; 6 Joint Research Center for Artificial Intelligence, King Fahd University of Petroleum & Minerals, Dhahran, Saudi Arabia; 7 Drug Supply Department, Hamad Medical Corporation, Doha, Qatar; 8 College of Pharmacy, QU Health, Qatar University, Doha, Qatar; 9 School of Pharmacy, Faculty of Medicine and Health, University of Sydney, Sydney, Australia; 10 Sydney Infectious Diseases Institute, University of Sydney, Sydney, Australia; 11 Department of Software Engineering, Faculty of Engineering and Information Technology, Al-Ruwad University, Taiz, Yemen

**Keywords:** Artificial intelligence, Machine learning, Precision dosing, Narrow therapeutic index drugs, Pharmacogenomics

## Abstract

**Background:**

Precision dosing of narrow therapeutic index drugs remains challenging because of substantial interindividual variability and the narrow margin between therapeutic efficacy and toxicity. Artificial intelligence (AI)–based approaches may improve individualized dosing by integrating complex clinical and pharmacogenomic data. This systematic review evaluated the predictive performance and applications of AI-based approaches for precision dosing in narrow therapeutic index drugs.

**Methods:**

A systematic review was conducted according to PRISMA guidelines. PubMed, Scopus, Cochrane Library, and IEEE Xplore were searched from inception to December 2025 for studies evaluating AI-based approaches for precision dosing of narrow therapeutic index drugs. Eligible studies included clinical investigations assessing predictive performance or clinical outcomes of AI-guided dosing models. Methodological quality and reporting completeness were evaluated using the PROBAST and TRIPOD tools, respectively.

**Results:**

Sixteen studies published between 2018 and 2025 were included, with most focusing on warfarin (*n* = 8) and tacrolimus (*n* = 5). Ensemble learning approaches, including random forest, XGBoost, extra trees regressor, heuristic stacking, and deep forest models, frequently demonstrated favorable predictive performance compared with conventional methods. Pharmacogenomic variables, particularly CYP2C9 and VKORC1 for warfarin and CYP3A5 for tacrolimus, were consistently identified as important predictors. The identified AI applications were primarily related to individualized dose prediction and therapeutic drug monitoring across anticoagulant, immunosuppressant, and antiepileptic therapies. However, substantial heterogeneity was observed in AI methodologies, target outcomes, validation strategies, and reported performance metrics. Most studies were retrospective and single-centre, while external validation and assessment of clinically meaningful outcomes remained limited.

**Conclusions:**

AI-based approaches show promising potential for improving precision dosing of narrow therapeutic index drugs, particularly when integrating pharmacogenomic and clinical variables. Nevertheless, current evidence is limited by methodological heterogeneity, inconsistent reporting, and insufficient external validation. Further prospective multicentre studies evaluating real-world clinical outcomes are needed before widespread clinical implementation can be recommended.

**Supplementary Information:**

The online version contains supplementary material available at https://doi.org/10.1186/s12911-026-03769-7.

## Introduction

Precision dosing is a major objective of contemporary pharmacotherapy, yet it remains particularly challenging for drugs with a narrow therapeutic index [1, 2]. These medications are characterized by a small margin between therapeutic benefit and toxicity, meaning that relatively minor deviations in dose or drug exposure may lead either to treatment failure or serious adverse effects [3, 4]. This challenge is especially relevant for drugs such as warfarin, tacrolimus, cyclosporine, valproic acid, and oxcarbazepine, where underexposure may lead to treatment failure, while overexposure can cause serious toxicity [5–7]. Consequently, narrow therapeutic index drugs often require close monitoring, frequent dose adjustments, and continued clinical supervision [8].

The complexity of dosing these drugs is driven by substantial variability in patient response [9]. Drug disposition and treatment effect are influenced by multiple interacting factors, including age, body weight, organ function, comorbidities, concurrent medications, disease severity, treatment adherence, and dynamic changes in clinical status over time [10–12]. For many patients, the same standard dose can produce markedly different therapeutic responses. This variability makes conventional one-size-fits-all dosing strategies inherently limited and reinforces the need for more individualized approaches that can account for multiple patient-specific variables simultaneously [13, 14].

In routine practice, dose optimization commonly relies on empirical starting regimens followed by reactive titration based on therapeutic drug monitoring, laboratory values, or clinical response [15]. Although widely used, this process can be slow and inefficient, particularly during treatment initiation or periods of clinical instability. Patients may remain outside the therapeutic range for prolonged periods before an effective and safe dose is reached, increasing the risk of preventable complications [16]. Traditional prediction tools based on linear regression or rule-based algorithms have improved standardization in some settings, but they are often constrained by assumptions of linearity, limited capacity to capture complex interactions, and reduced adaptability to evolving patient conditions [17, 18]. In addition, many existing models have shown variable performance when applied across different populations or healthcare environments [19].

Artificial intelligence (AI) has emerged as a promising strategy to overcome these limitations. AI is an overarching field that encompasses machine learning techniques, including deep learning, which uses multilayer neural networks to model complex patterns in data. Machine learning and deep learning methods can analyze large, multidimensional datasets and capture complex nonlinear relationships among clinical variables, laboratory parameters, medication history, and longitudinal treatment data [20, 21]. In precision dosing, these systems aim to generate individualized dose recommendations that are more accurate, adaptive, and scalable than conventional approaches [22]. A broad range of model architectures has been investigated, including ensemble methods such as random forest, XGBoost, and extra trees, deep learning approaches such as long short-term memory (LSTM) networks and deep neural networks, as well as kernel-based methods such as support vector machines [23, 24]. Emerging evidence suggests that AI-based approaches may improve predictive performance across several narrow therapeutic index drugs [25]. These findings suggest that AI may offer meaningful advantages in therapeutic individualization, particularly for medications where conventional titration is difficult or resource-intensive [26].

Despite these promising advances, several important gaps remain in the current evidence base [27]. The published literature is highly heterogeneous with respect to study design, sample size, patient populations, predictors, validation strategies, and reported outcomes. Most studies are retrospective and conducted in single centers, whereas robust external validation remains limited. Moreover, favorable predictive metrics such as mean absolute error (MAE), root mean square error (RMSE), coefficient of determination (R²), and percentage within ± 20% of target dose (PW20) do not necessarily translate into improved patient outcomes [28]. Evidence linking AI-guided dosing to clinically meaningful outcomes, including time in therapeutic range, major bleeding, graft rejection, seizure control, and healthcare utilization, remains limited [29].

Given the growing need for individualized therapy and the rapid expansion of AI in healthcare, a rigorous synthesis of the available evidence is needed. Therefore, this systematic review aimed to evaluate the current evidence regarding the use of AI-based approaches for precision dosing of narrow therapeutic index drugs, particularly their predictive performance and applications across different therapeutic areas. The findings of this systematic review may help guide future research and support the integration of AI-driven dosing strategies into clinical practice.

## Methods

### Study design and registration

This study was designed as a systematic review to evaluate the role of AI in precision dosing of narrow therapeutic index drugs. The review was conducted in accordance with the Preferred Reporting Items for Systematic Reviews and Meta-Analyses (PRISMA) statement [30]. The review protocol was prospectively registered in the International Prospective Register of Systematic Reviews (PROSPERO; CRD420261360468) [31]. A completed PRISMA checklist is provided in the Supplementary File 1.

### Search strategy

A systematic literature search was conducted in PubMed, Scopus, Cochrane Library, and IEEE Xplore from database inception to December 2025 to identify studies evaluating the use of AI in precision dosing of narrow therapeutic index drugs. The search strategy combined controlled vocabulary terms, where applicable, and free-text keywords related to “artificial intelligence, machine learning, deep learning, drug dosing, dose regimens, dose adjustment, dose optimization, individualized dosing, personalized dosing, dose prediction, and dose calculation”. Because terminology describing narrow therapeutic index drugs varies across studies and databases, eligibility assessment was additionally used to confirm the inclusion of studies involving relevant drugs. Boolean operators (“AND” and “OR”) were applied according to the requirements of each database to optimize search sensitivity and specificity. Only studies published in English were included. Detailed search strategies for all databases are provided in the Supplementary File 2 (Table S1).

### Eligibility criteria and study selection

Study eligibility was predefined using the PICOS framework. Eligible studies included adult or pediatric populations receiving narrow therapeutic index drugs requiring individualized dose optimization. Narrow therapeutic index drugs were defined a priori based on regulatory classifications and published literature describing medications with a narrow margin between therapeutic efficacy and toxicity, where relatively small changes in systemic exposure may result in treatment failure or adverse drug reactions. Eligible studies involving AI or machine learning models for individualized dosing of recognized Narrow therapeutic index drugs were considered, irrespective of the specific therapeutic area.

The intervention of interest was the application of AI-based methods for dose prediction, dose optimization, or therapeutic concentration forecasting. Comparators included conventional dosing strategies, clinician-guided dosing, traditional statistical approaches, pharmacokinetic methods, Bayesian models, or no comparator in model development studies. Primary outcomes were predictive performance metrics, including MAE, RMSE, R², and PW20, while secondary outcomes included clinically relevant endpoints when reported. Randomized trials, prospective and retrospective cohort studies, observational studies, and external validation studies using real patient data were eligible. Reviews, editorials, conference abstracts without sufficient data, animal studies, in vitro studies, and studies lacking extractable outcomes were excluded. The detailed inclusion and exclusion criteria according to each PICOS element are provided in Supplementary File 2 (Table S2).

All retrieved records were imported into Rayyan, an AI-assisted systematic review platform, and duplicate records were removed. Rayyan’s artificial intelligence–assisted features were used to facilitate citation management and the screening process; however, all title and abstract screening, full-text eligibility assessment, and final study inclusion decisions were performed independently by the reviewers according to the predefined eligibility criteria. Study selection was conducted in two phases: (1) title and abstract screening and (2) full-text eligibility assessment. Screening was performed independently by two reviewers (AA and MA), and disagreements were resolved through discussion with involvement of a third reviewer (SDA) when required.

### Data extraction

Data extraction was conducted independently by two reviewers using a standardized and piloted data collection form developed in Microsoft Excel. Any discrepancies were resolved through discussion, with consultation from a third reviewer when necessary. Extracted data included study characteristics (first author, publication year, country, and study design), patient and population characteristics, sample size, narrow therapeutic index drug evaluated, clinical setting, and details of the AI approach, including model architecture, input variables, comparator methods, and validation strategy.

Outcome data extracted from eligible studies included predictive performance metrics such as MAE, RMSE, R², PW20, accuracy, sensitivity, specificity, and area under the receiver operating characteristic curve, where applicable. These metrics were calculated according to the prediction target specified in each study, including maintenance dose, dose requirement, trough concentration, plasma concentration, drug clearance, or international normalized ratio (INR), depending on the clinical application. Because the prediction targets differed across studies, direct quantitative comparison of absolute performance metrics was not considered appropriate. Clinically relevant outcomes, including time in therapeutic range, adverse drug events, graft rejection, seizure control, dose attainment, and healthcare utilization, were also extracted when reported. Where multiple eligible AI models were presented within a single study, data from all relevant models were extracted.

### Risk of bias assessment

Methodological quality of the included studies was assessed independently by two reviewers (AA and MA) using the Prediction model Risk of Bias Assessment Tool (PROBAST), which is recommended for studies that develop, validate, or update multivariable prediction models. PROBAST evaluates potential sources of bias across four domains: participants, predictors, outcomes, and analysis [32]. Each signalling question was judged as “yes,” “probably yes,” “probably no,” “no,” or “no information,” leading to domain-level judgments of low, high, or unclear risk of bias, followed by an overall risk-of-bias judgment for each study according to PROBAST guidance [32]. Any disagreements between reviewers were resolved through discussion, with consultation from a third reviewer when necessary. Risk-of-bias assessments were used to inform the interpretation of study findings and identify methodological limitations across the evidence base. No studies were excluded based on risk-of-bias judgments.

In addition to methodological appraisal, reporting quality was evaluated using the Transparent Reporting of a multivariable prediction model for Individual Prognosis Or Diagnosis (TRIPOD) checklist [33]. Each study was assessed against the 22-item TRIPOD reporting framework, and a summary score (0–22) was calculated based on the number of adequately reported items, with higher scores indicating more complete and transparent reporting. TRIPOD scores were used descriptively to compare reporting completeness across studies and did not replace the formal risk-of-bias assessment.

### Data synthesis

A narrative synthesis was undertaken for all included studies and was structured according to drug class, clinical application, and AI methodology. Study characteristics, model architectures, validation strategies, predictors, comparators, and reported outcomes were summarized descriptively. Extracted data were organized and managed using Microsoft Excel. The original review protocol anticipated quantitative synthesis where sufficient methodological homogeneity was available. However, after study selection and data extraction, substantial heterogeneity across studies in terms of study design, patient populations, target drugs, AI methodologies, outcome definitions, performance metrics, and reporting approaches, quantitative synthesis using meta-analysis was not considered appropriate. Therefore, findings were synthesized qualitatively through comparative analysis of model performance, methodological characteristics, and reported clinical outcomes across studies. Risk-of-bias assessments were conducted using the relevant appraisal tools, and graphical visualizations of risk-of-bias judgments were generated using appropriate software packages.

## Results

### Study selection

The database search identified a total of 393 records, including 166 from Scopus, 131 from PubMed, 76 from the Cochrane Library, and 20 from IEEE Xplore. After removal of 43 duplicate records, 350 unique records remained for title and abstract screening. Of these, 318 records were excluded as clearly irrelevant to the review question.

**Fig. 1:**
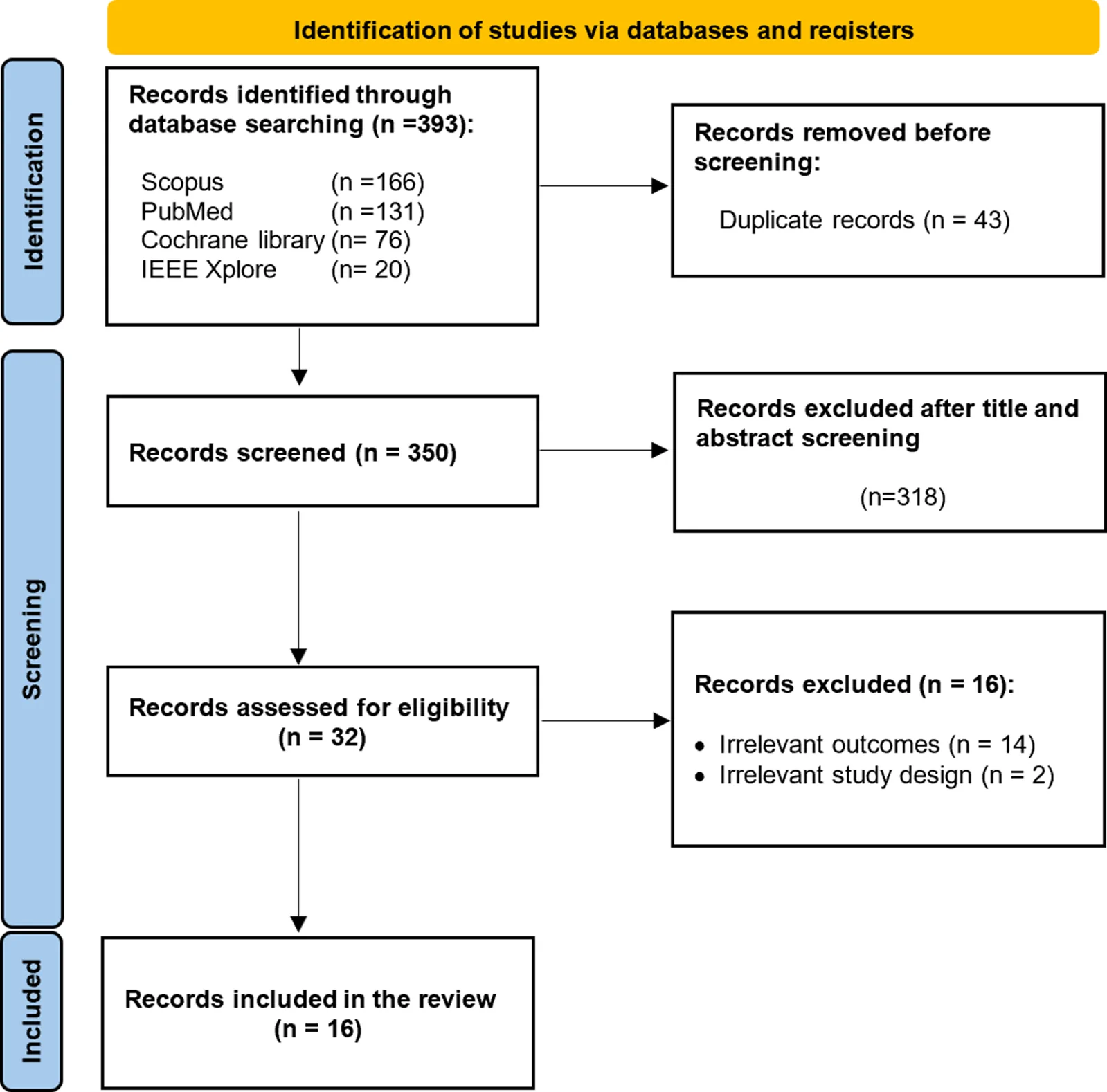
PRISMA diagram of the study selection process

A total of 32 full-text articles were assessed for eligibility. Following full-text review, 16 studies were excluded, primarily because of irrelevant outcomes (*n* = 14) or ineligible study design (*n* = 2). Ultimately, 16 studies met the predefined eligibility criteria and were included in the systematic review. The study selection process is summarized in Fig. 1. The detailed screening checklist, including eligibility decisions for all records assessed during phase 1 and phase 2 screening, is provided in the Supplementary File 2 (Table S3).

### Characteristics of included studies

A total of 16 studies published between 2018 and 2025 were included in the final review. The studies were conducted predominantly in Asia, especially China [34–43], with other studies from South Korea [44], India [45], Bahrain [46], South Africa [47], Puerto Rico [48], and Canada [49]. Sample sizes ranged from 108 participants [43] to 10,362 participants [47]. Most studies used retrospective designs [34, 36, 38–41, 43, 45–48, 50], while two were prospective [42, 49] and two were mixed/prospective designs [35, 44].

Warfarin was the most frequently investigated drug (*n* = 8), followed by tacrolimus (*n* = 5), while three studies evaluated other narrow therapeutic index drugs, namely valproic acid, oxcarbazepine, and a combined tacrolimus/cyclosporine model. Warfarin studies primarily targeted INR-guided dose prediction across therapeutic ranges of 1.5–2.5, 1.6–2.5, or 2.0–3.0 [34–36, 38, 44, 47, 48, 50]. Tacrolimus studies focused on trough concentration (5–10 ng/mL), stable dose (mg/day), dose requirement (mg/kg), or drug clearance (L/h) [39–41, 45, 49]. The remaining studies predicted plasma concentrations of valproic acid (50–100 mg/L), oxcarbazepine (50–100 µg/mL), or trough concentrations for combined tacrolimus/cyclosporine therapy (5–20 ng/mL) [42, 43, 46].

A broad spectrum of AI approaches was identified. Kernel-based methods included support vector machine (SVM) radial models for warfarin prediction [34, 47]. Deep learning models included LSTM networks for warfarin prediction and non-greedy optimization-based artificial neural network (NGO-BPANN) models for oxcarbazepine prediction [35, 43]. Ensemble learning methods were the most common overall and included random forest, heuristic stacking, gradient boosting machine (GBM), extra trees regressor, extreme gradient boosting (XGBoost), and deep forest models applied across warfarin, tacrolimus, and valproic acid studies [39, 40, 42, 44, 45, 48, 50]. Hybrid and statistical models included genetic algorithm–back propagation neural network (GA-BPNN), multivariate adaptive regression splines (MARS), linear regression, and hybrid population pharmacokinetic–least absolute shrinkage and selection operator (PPK-LASSO) approaches [36, 38, 41, 46]. Overall, ensemble-based models predominated, particularly in warfarin and tacrolimus studies, whereas hybrid and kernel-based approaches were frequently used in pharmacogenomic-guided dosing settings. More details are provided in Table 1.

**Table 1:** Characteristics of the included studies evaluating artificial intelligence–based precision dosing of narrow therapeutic index drugs

Author, Year	Drug (Class)	Country	Study Type	Sample Size (*N*)	AI Model	Comparator	Target Metric & Therapeutic Range	TRIPOD Score (/22)
**Warfarin (*n* = 8)**								
Guo et al., 2025 [34]	Warfarin (Anticoagulant)	China	Retrospective	413	SVM Radial (Kernel-based ML)	CNN (Deep Learning)	INR (1.5–2.5)	19
Kuang et al., 2022 [35]		China	Mixed	782	LSTM (Deep Learning)	MAPB Algorithm (Clinical Algorithm)	INR (1.5–2.5)	15
Li et al., 2018 [36]		China	Retrospective	289	GA-BPNN (Hybrid Neural Network)	IWPC / MLR (Statistical Model)	INR (1.5–2.5)	19
Wang et al., 2023 [37]		China	Retrospective	641	Heuristic-Stacking (Ensemble Learning)	Non-stacking (Ensemble Learning)	INR (1.6–2.5)	18
Ma et al., 2021 [38]		China	Retrospective	5,741	LR (Statistical Model)	None Reported	INR (1.5–2.5)	19
Nguyen et al., 2021 [44]		South Korea	Mixed	650	Gradient Boosting Machine (Ensemble Learning)	LR (Statistical Model)	INR (2.0–3.0)	19
Truda et al., 2021 [47]		South Africa	Retrospective	10,362	SVM Radial (Kernel-based ML)	LR / Naïve Mode (Statistical Model)	INR (2.0–3.0)	13
Roche-Lima et al., 2020 [48]		Puerto Rico	Retrospective	190	Random Forest Regression (Ensemble Learning)	Regression Models (Statistical / ML)	INR (2.0–3.0)	18
**Tacrolimus** (***n* = 5)**								
Min et al., 2025 [39]	Tacrolimus (Immunosuppressant)	China	Retrospective	288	GA Deep Forest (Ensemble Learning)	GBDT (Machine Learning)	Trough Concentration (5–10 ng/mL)	19
Fu et al., 2022 [40]		China	Retrospective	2,551	Extra Trees Regressor (Ensemble Learning)	Multiple ML Models (Machine Learning)	Dose Requirement(mg/kg)	20
Huang et al., 2022 [41]		China	Retrospective	139	Hybrid PPK-Lasso (PK/Regularized Regression)	Extra Trees (Ensemble Learning)	Clearance (L/h)	18
Raval et al., 2025 [45]		India	Retrospective	1,045	XGBoost (Ensemble Learning)	XGBoost (Ensemble Learning)	Stable Dose (mg/day)	19
Min et al., 2022 [49]		Canada	Prospective	777	Lasso (Regularized Regression)	LR (Statistical Model)	Trough Concentration (5–10 ng/mL)	20
**Other narrow therapeutic index Drugs** (***n*** ** = 3)**								
Cai et al., 2025 [42]	Valproic Acid (Antiepileptic)	China	Prospective	275	Random Forest (Ensemble Learning)	GLM (Statistical Mode	Plasma Concentration (50–100 mg/L)	17
Xu et al., 2024 [43]	Oxcarbazepine (Antiepileptic)	China	Retrospective	108	NGO-BPANN (Deep Learning Neural Network)	PopPK Model (Pharmacokinetic Model)	Plasma Concentration (50–100 µg/mL)	18
Sridharan et al., 2023 [46]	TAC/Cyclosporine (Immunosuppressant)	Bahrain	Retrospective	120	MARS (Statistical/Hybrid Model)	SVM / ANN / GLM (Multiple Models)	Trough Concentration (5–20 ng/mL)	16

Abbreviations: AI, artificial intelligence; ANN, artificial neural network; CNN, convolutional neural network; GA, genetic algorithm; GBDT, gradient boosting decision tree; GLM, generalized linear model; INR, international normalized ratio; LASSO, least absolute shrinkage and selection operator; LR, linear regression; LSTM, long short-term memory; MARS, multivariate adaptive regression splines; ML, machine learning; MLR, multiple linear regression; NGO-BPANN, non-greedy optimization-based artificial neural network; NTI, narrow therapeutic index; PK, pharmacokinetic; PopPK, population pharmacokinetic; RF, random forest; SVM, support vector machine; TAC, tacrolimus

### Performance of AI-based precision dosing models across drug classes

Across the included studies, model performance varied according to drug class, predictor availability, study population, and modelling strategy; however, several recurring trends were observed (Table 2). Interpretation of predictive performance metrics should be made in the context of each study’s prediction target. Although MAE, RMSE, R², and PW20 were commonly reported across studies, these metrics were calculated using different target variables, including predicted versus observed maintenance dose, dose requirement, plasma or trough drug concentration, drug clearance, or INR. Consequently, absolute values of these metrics are not directly comparable across therapeutic areas or prediction tasks, and model performance was interpreted relative to the comparator evaluated within each individual study.

**Table 2:** Summary of reported performance outcomes of AI-based precision dosing models across included studies

Author, Year	Genetic Predictors	Prediction Target				Performance vs. Comparator
		MAE	RMSE	*R*²	PW20 (%)	
**Warfarin (*n* = 8)**						
Guo et al., 2025 [34]	CYP2C9, VKORC1	0.14 mg/day	NR	0.98	93.83	Promising predictive performance
Kuang et al., 2022 [35]	CYP2C9, VKORC1	0.50 INR	0.80 INR	NR	53.93	Promising predictive performance
Li et al., 2018 [36]	CYP2C9, VKORC1	0.38 mg/day	0.66 mg/day	NR	58.70–62.90	Promising predictive performance
Wang et al., 2023 [37]	CYP2C9, VKORC1	0.11 mg/day	0.18 mg/day	0.87	73.44	Promising predictive performance
Ma et al., 2021 [38]	NR	0.41 mg/day	NR	NR	75.60	Mixed findings
Nguyen et al., 2021 [44]	CYP2C9, VKORC1	0.64 mg/day	0.90 mg/day	0.53	73.85	Comparable
Truda et al., 2021 [47]	CYP2C9, VKORC1	1.56 mg/day	NR	NR	34.46	Comparable to LR; superior to naïve model
Roche-Lima et al., 2020 [48]	Multiple PGx variants (CYP2C9, VKORC1, CYP4F2, ABCB1, others)	0.68 mg/day	NR	NR	80.56	Promising predictive performance
**Tacrolimus** (***n* = 5)**						
Min et al., 2025 [39]	CYP3A5	NR	0.78 mg/day	0.23	NR	Promising predictive performance
Fu et al., 2022 [40]	CYP3A5, CYP3A4	NR	NR	0.87	93.13	Promising predictive performance
Huang et al., 2022 [41]	CYP3A5, CTLA4	1.51 L/h	0.21†	0.42	NR	Promising predictive performance
Raval et al., 2025 [45]	CYP3A5	0.41 mg/day	0.062†	0.83	87.60	Promising predictive performance
Min et al., 2022 [49]	CYP3A5, rs12333983, rs12957142, others	0.55 mg	0.55 mg/day	0.60	NR	Promising predictive performance
**Other NTI Drugs** (***n*** ** = 3)**						
Cai et al., 2025 [42]	CYP2C19, CYP2C9, UGT2B7	10.57 mg/L	13.84 mg/L	0.39	NR	Promising predictive performance
Xu et al., 2024 [43]	SCN1A rs2298771, SCN2A rs17183814	NR	NR	0.94‡	NR	Promising predictive performance
Sridharan et al., 2023 [46]	CYP3A53, CYP3A422, ABCB1, PPAR-α, POR*28	1.30 / 73.3 mg/day	1.50 / 91.7	0.30–0.70	9.20 / 21.20	Mixed findings

Abbreviations: MAE = Mean Absolute Error; RMSE = Root Mean Square Error; R² = Coefficient of Determination; PW20 = Percentage of predictions within ± 20% of observed value; NR = Not Reported; PGx = Pharmacogenomic; SNP = Single nucleotide polymorphism; INR = International Normalized Ratio; LR = Linear Regression. Units vary according to study endpoint (e.g., mg/day, INR, mg/L, L/h). †Reported as published; interpret with caution due to scale/metric differences. ‡Pearson’s *r* reported instead of R²

Note: Interpretations are based on findings reported within individual studies and should not be considered direct comparisons across therapeutic areas, study populations, or outcome measures

For warfarin dosing, machine learning and ensemble-based approaches frequently demonstrated favorable performance relative to conventional comparators within individual studies. One of the highest reported performances was observed for support vector regression in a Chinese cohort (R² = 0.98; PW20 = 93.83%) [34]. Other models with favorable results included LSTM networks [35], hybrid neural network approaches integrating back-propagation with genetic algorithms [36], random forest models [48], and heuristic-stacking frameworks [37]. In contrast, some studies reported mixed or only comparable benefit versus standard approaches, including feedforward neural networks [38] and gradient boosting methods [44], suggesting that increased algorithmic complexity does not uniformly translate into better clinical prediction.

For tacrolimus, ensemble-based models again appeared promising, particularly in studies integrating clinical and pharmacogenomic variables. Extra Trees Regressor achieved strong reported performance (R² = 0.87; PW20 = 93.13%) and demonstrated more favorable performance than several competing machine learning approaches within the same study [40]. Similarly, the Dose Optimal framework combining LightGBM, CatBoost, and Random Forest demonstrated robust performance (R² = 0.83; PW20 = 87.60%) and showed more favorable performance than standalone XGBoost within that study [45]. Deep Forest with genetic optimization also showed favorable findings [39], whereas regularized regression approaches such as LASSO demonstrated more modest or variable performance across studies [41, 49].

Among other narrow therapeutic index drugs, the evidence base was smaller but still informative. For oxcarbazepine/monohydroxy derivative prediction, a hybrid neural network model demonstrated high reported discrimination (R² ≈ 0.94), with more favorable performance than the population pharmacokinetic model evaluated in the same study [43]. In contrast, valproic acid prediction using random forest showed moderate explanatory performance (R² = 0.39), although it demonstrated more favorable performance than generalized linear modelling within that study [42]. A combined tacrolimus/cyclosporine study reported mixed findings, potentially reflecting the challenge of modelling two pharmacokinetically distinct drugs within a single framework [46].

Several predictors were repeatedly associated with dose requirements or drug exposure, although their relative importance differed by therapeutic area. For warfarin, recurrent predictors included CYP2C9, VKORC1, age, body size measures, and interacting medications such as amiodarone [34–37, 44, 47, 48]. For tacrolimus, commonly reported predictors included CYP3A5, body weight, hematocrit, albumin, and time after transplantation [39–41, 45, 49]. For antiepileptic drugs, influential variables included therapeutic drug monitoring values and genes related to metabolism or transport, such as CYP2C19, UGT2B7, ABCB1, and sodium-channel variants [42, 43]. Collectively, these findings suggest that AI-guided dosing models may achieve the most favorable predictive performance when integrating biological factors with routinely collected clinical data.

### Comparison of AI model families and conventional approaches

Across the included studies, the evaluated prediction models could be broadly classified into five methodological categories: ensemble learning (e.g., Random Forest, Gradient Boosting Machine, XGBoost, Extra Trees Regressor, Deep Forest, and heuristic stacking), deep learning (e.g., LSTM and convolutional neural network [CNN]), kernel-based machine learning (support vector machine [SVM]), hybrid models combining multiple computational techniques (e.g., GA-BPNN and hybrid population pharmacokinetic–LASSO models), and conventional statistical approaches including linear regression, generalized linear models, and regularized regression (Table 1). Across studies, AI-based models were most frequently compared with conventional statistical models, pharmacokinetic models, clinical dosing algorithms, Bayesian approaches, or alternative machine learning algorithms rather than against a single universal comparator. Consequently, model performance should be interpreted relative to the comparator evaluated within each individual study rather than across studies.

Among the evaluated AI model families, ensemble learning approaches demonstrated the most consistent favorable performance across different therapeutic applications. Random Forest, Gradient Boosting Machine, heuristic stacking, Extra Trees Regressor, Deep Forest, and XGBoost-based frameworks generally achieved superior or comparable predictive performance relative to conventional statistical models or alternative machine learning approaches in studies involving warfarin, tacrolimus, and valproic acid dosing, particularly when pharmacogenomic and routinely collected clinical variables were incorporated into the prediction models [37, 39, 40, 42, 44, 45]. These findings suggest that ensemble learning methods may provide greater robustness when modelling the complex nonlinear relationships underlying individualized dose prediction.

Deep learning models, including LSTM and CNN architectures, also demonstrated promising predictive capability, particularly for warfarin and oxcarbazepine prediction [34, 35, 43]. However, the available evidence for deep learning remained comparatively limited, with relatively few studies evaluating these approaches. Likewise, kernel-based machine learning, represented primarily by support vector machine models, achieved excellent predictive performance for warfarin dose prediction in selected cohorts [34, 47], although evidence was confined to a small number of studies. Hybrid approaches, including GA-BPNN and hybrid population pharmacokinetic–LASSO models, generally improved predictive performance compared with conventional regression or pharmacokinetic models but showed more variable performance depending on the prediction task and available predictors [36, 41].

When compared with traditional approaches, AI-based models generally demonstrated superior or at least comparable predictive performance within individual studies. Conventional comparators included linear regression models, generalized linear models, pharmacokinetic models, clinical dosing algorithms, and Bayesian or population pharmacokinetic frameworks [35, 36, 38, 39, 41–43, 47, 48]. Nevertheless, the magnitude of performance improvement varied considerably according to drug class, prediction target, input variables, study population, and validation strategy. Because the included studies differed substantially in methodological design and outcome reporting, no single AI model family can currently be considered universally superior. Rather, the available evidence suggests that ensemble learning approaches have demonstrated the most consistent advantages across multiple therapeutic settings, whereas further external validation and prospective clinical evaluation are needed before definitive recommendations regarding specific AI model families can be made.

### Generalizability and evidence gaps

Despite encouraging results, several limitations may restrict the generalizability and clinical translation of current AI-based precision dosing models. External validation was reported in only a subset of studies [35, 40, 43, 45, 47–49]. In contrast, many models were developed using single-center or country-specific cohorts, creating uncertainty regarding performance in broader or more diverse populations and healthcare systems [41, 42, 46]. This issue is particularly relevant for narrow therapeutic index drugs, where prescribing practices, monitoring intensity, and patient characteristics may vary substantially across regions and institutions.

Several studies also relied on relatively small sample sizes, increasing the risk of overfitting and unstable performance estimates, particularly when complex machine learning models were trained using high-dimensional predictor sets [41, 42]. In addition, reporting standards were inconsistent across studies. Some investigations provided multiple performance metrics, whereas others reported only selected outcomes, limiting direct comparison and evidence synthesis. Outcome definitions also varied substantially across therapeutic areas. This was especially evident for tacrolimus, where models targeted trough concentration, stable dose, dose requirement, or clearance rather than a single standardized endpoint [39–41, 45, 49]. Similar heterogeneity was observed across other drug classes, with some studies focusing on maintenance dose and others on pharmacokinetic exposure measures. Such variability complicates cross-study comparison and limits immediate implementation in routine clinical practice.

### Quality assessment

**Fig. 2:**
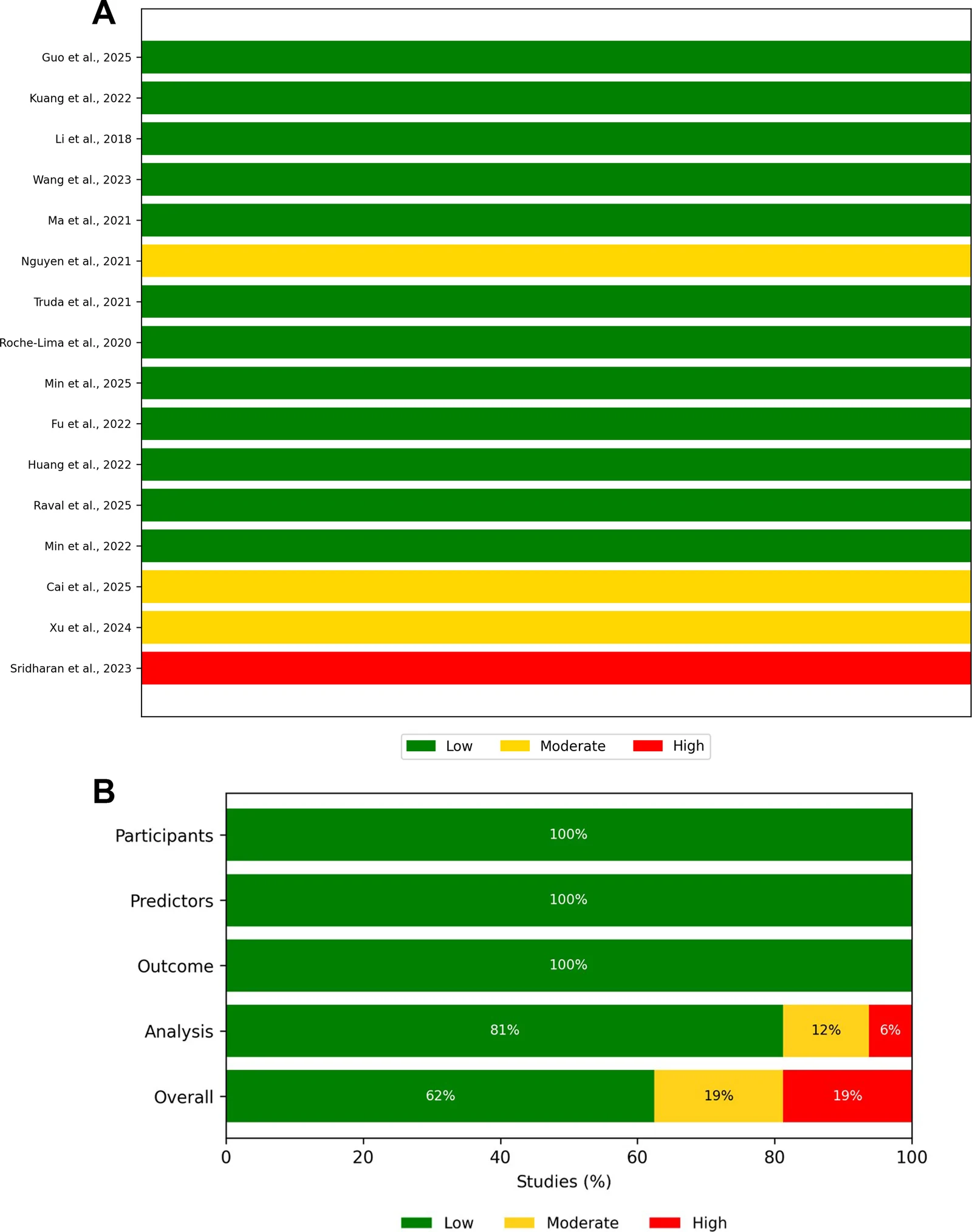
**A**. Overall risk of bias assessment of included studies using the PROBAST tool. **B **Summary of PROBAST risk-of-bias judgments by domain

Methodological quality assessed using the PROBAST tool indicated that most included studies had an overall low risk of bias [34–36, 38–40, 45, 47–50]. A smaller number of studies were judged as having moderate risk of bias, mainly due to limited sample size, unclear validation procedures, incomplete calibration reporting, or potential overfitting [42–44]. One study was judged as having high risk of bias because of the lack of independent external validation [46]. Across studies, the most frequent concerns arose in the analysis domain, whereas the participants, predictors, and outcome domains were generally rated as low risk because cohorts, predictors, and therapeutic monitoring outcomes were usually well defined. Applicability concerns were mostly low to moderate, with some limitations related to single-centre cohorts, ethnicity-specific populations, or restricted clinical settings. Figure 2A presents domain-specific and overall PROBAST judgments across the included studies, whereas Fig. 2B summarizes the proportion of studies judged as having low, moderate, and high risk of bias across each PROBAST domain and the overall assessment. Detailed PROBAST assessments, signalling-question responses, and justifications are provided Supplementary File 3.

Reporting quality assessed using the TRIPOD checklist was generally moderate to high, with scores ranging from 13 to 20 out of 22 (Table 1). Most studies scored between 18 and 20, indicating relatively complete reporting of study design, predictors, model development, and performance measures. The highest scores (20/22) were observed in two tacrolimus studies [40, 49], while several studies achieved 19/22 [34, 36, 38, 39, 44, 45]. Lower scores were reported for three studies [35, 46, 47]. The most commonly underreported items were sample size justification, handling of missing data, calibration assessment, and full model specification. Overall item-level TRIPOD assessments and scoring details are provided in Supplementary File 3.

## Discussion

### Principal findings

This systematic review evaluated the current evidence regarding the use of AI-based approaches for precision dosing of narrow therapeutic index drugs. Overall, the findings suggest that AI-driven models demonstrate promising predictive performance across several therapeutic areas, particularly for warfarin and tacrolimus. Ensemble learning methods, including random forest, XGBoost, extra trees regressor, heuristic stacking, and deep forest models, were among the most consistently high-performing approaches across included studies. Additionally, pharmacogenomic variables repeatedly emerged as important contributors to dose prediction, particularly for drugs with substantial interindividual variability in metabolism and exposure. However, direct comparison of predictive performance across therapeutic areas should be interpreted with caution because the included studies differed substantially in target outcomes, populations, predictors, and performance metrics. Nevertheless, the current evidence base remains heterogeneous, predominantly retrospective, and limited by inconsistent reporting and insufficient external validation, which collectively restrict immediate clinical implementation.

Warfarin was the most extensively studied drug in the included literature, reflecting its longstanding role as one of the most clinically challenging narrow therapeutic index medications. Warfarin dosing is highly variable because of complex interactions among genetic, demographic, dietary, pharmacokinetic, and pharmacodynamic factors [51]. Consistent with established pharmacogenomic evidence, CYP2C9 and VKORC1 were recurrently identified as dominant predictors across most warfarin models [52]. Several AI-based approaches demonstrated favorable predictive performance relative to conventional regression-based methods within individual studies, particularly support vector regression, heuristic stacking frameworks, random forest models, and long short-term memory (LSTM) networks. These findings are biologically plausible because nonlinear machine learning algorithms are capable of capturing complex interactions and temporal relationships that may not be adequately addressed using traditional linear modelling approaches [53–55]. These advantages may be particularly relevant for warfarin, where multiple interacting variables influence dose-response relationships. The favorable performance of ensemble learning methods may also reflect their ability to reduce variance and improve generalization by combining multiple predictive learners within a single framework [56].

Tacrolimus represented the second most frequently investigated drug class and demonstrated similar trends favoring AI-guided dosing approaches. Tacrolimus pharmacokinetics are particularly difficult to predict because of substantial interpatient variability related to CYP3A5 genotype, transplantation status, organ function, hematocrit, albumin levels, interacting medications, and time-dependent physiologic changes following transplantation [57, 58]. Across the included studies, models integrating both clinical and pharmacogenomic data generally achieved superior predictive performance compared with conventional approaches. Ensemble-based algorithms such as Extra Trees Regressor, Deep Forest, and hybrid gradient boosting frameworks showed especially favorable results. These findings support the growing recognition that integrating genomic information into AI-based dosing frameworks may improve individualized immunosuppressive therapy [59, 60]. However, important heterogeneity remained across tacrolimus studies, particularly regarding target outcomes, which included trough concentration, dose requirement, stable dose, and drug clearance.

Beyond warfarin and tacrolimus, evidence for other narrow therapeutic index drugs, including valproic acid, oxcarbazepine, and combined tacrolimus/cyclosporine models, was more limited but nevertheless informative. AI-based approaches generally outperformed traditional pharmacokinetic or statistical comparators within individual studies, although predictive performance varied substantially. For example, the oxcarbazepine neural network model demonstrated strong reported discrimination, whereas valproic acid models achieved only moderate explanatory performance despite outperforming generalized linear modelling approaches. These findings suggest that AI-driven precision dosing may be applicable beyond anticoagulants and immunosuppressants, although the current evidence remains insufficient to draw definitive conclusions across broader narrow therapeutic index drug classes [61, 62].

### Clinical implications of AI-guided precision dosing

An important finding of this review is that most studies primarily evaluated predictive accuracy rather than clinically meaningful patient outcomes. Although improved dose prediction may theoretically reduce toxicity, improve therapeutic target attainment, and enhance treatment safety, evidence demonstrating direct clinical benefit remains limited [63]. Notably, whether improved predictive accuracy translates into benefits beyond existing pharmacometric approaches remains unclear. This gap is particularly important because successful clinical implementation depends not only on predictive performance but also on demonstrable improvements in patient outcomes. In addition, several practical challenges may affect the adoption of AI-guided dosing systems in routine clinical care [64]. Many advanced machine learning models remain difficult to interpret, potentially limiting clinician trust and acceptance in high-risk therapeutic settings [65]. The growing interest in explainable AI may help address these concerns by improving transparency and supporting clinician understanding of model-generated recommendations. Furthermore, integration with electronic health record systems, therapeutic drug monitoring workflows, and pharmacogenomic infrastructure remains complex and resource dependent [66]. Additional challenges include regulatory oversight, validation requirements, and questions regarding accountability when AI-generated recommendations influence clinical decision-making. These considerations suggest that, despite promising predictive performance, current AI-guided dosing systems should still be considered investigational in many clinical settings.

### Methodological considerations

Several methodological concerns were identified across the included studies. Most investigations used retrospective single-center designs, which may limit external validity and increase the risk of selection bias and overfitting [63]. Sample sizes also varied substantially, with some studies using relatively small cohorts despite applying highly complex machine learning architectures. External validation was performed in only a limited number of studies, reducing confidence regarding model generalizability across different populations and healthcare systems [64]. Moreover, reporting standards were inconsistent, particularly regarding calibration assessment, handling of missing data, and complete model specification. Considerable heterogeneity was also observed in outcome definitions and reported performance metrics. This variability prevented quantitative synthesis using meta-analysis and necessitated a narrative comparative approach.

### Strengths, limitations, and future directions

This review has some strengths. The study was conducted according to PRISMA recommendations and prospectively registered in PROSPERO. Multiple electronic databases were systematically searched, and methodological quality was evaluated using both PROBAST and TRIPOD frameworks. Furthermore, the review included multiple narrow therapeutic index drug classes and a broad range of AI methodologies, providing a comprehensive overview of current developments in AI-guided precision dosing. Nevertheless, several limitations should be acknowledged. Only English-language studies were included, which may have introduced language bias. The inability to conduct meta-analysis limited quantitative comparison across studies. In addition, most included studies focused on surrogate prediction metrics rather than patient-important clinical outcomes, limiting conclusions regarding the real-world clinical benefit of AI-guided dosing strategies. Publication bias also cannot be excluded, as studies reporting favorable predictive performance may be more likely to be published than studies with neutral or negative findings. Furthermore, because AI research evolves rapidly, additional studies and newer model architectures may emerge after completion of the review. Although several major narrow therapeutic index drug classes were represented, the available evidence was identified primarily for warfarin, tacrolimus, valproic acid, oxcarbazepine, and cyclosporine, whereas no eligible AI-based individualized dosing studies meeting the inclusion criteria were identified for other commonly recognized NTI drugs, such as digoxin, lithium, phenytoin, or theophylline. This highlighs the need for further research in other clinically important narrow therapeutic index drugs. Future research should also prioritize prospective multicentre validation studies with standardized reporting frameworks and clinically meaningful endpoints [65]. Greater emphasis is needed on external validation, calibration assessment, explainable AI methodologies, and real-world implementation studies evaluating patient outcomes rather than prediction accuracy alone [66, 67]. Standardization of outcome definitions and reporting metrics would also improve comparability across studies and strengthen evidence synthesis in future reviews.

## Conclusion

This systematic review demonstrates that AI–based approaches have shown promising potential for improving precision dosing of narrow therapeutic index drugs, particularly for warfarin and tacrolimus. Ensemble learning and hybrid machine learning models frequently achieved favorable predictive performance, especially when integrating pharmacogenomic and clinical variables. However, the current evidence base is limited by substantial methodological heterogeneity, the predominance of retrospective single-center studies, inconsistent reporting standards, limited external validation, and the lack of clinically meaningful outcome evaluation. Consequently, although AI-guided precision dosing represents an important area of investigation in personalized pharmacotherapy, the available evidence is not yet sufficient to support its routine clinical implementation. Further prospective multicenter studies with standardized methodologies, robust external validation, and evaluation of real-world clinical outcomes are required before AI-guided dosing can be recommended for widespread clinical practice.

## Supplementary Information

Below is the link to the electronic supplementary material.

Supplementary Material 1

Supplementary Material 2

Supplementary Material 3

## Data Availability

All data presented in the current work are available in the text and supplementary materials and can also be obtained from the authors upon request.

## Abbreviations

ABCB1: ATP-binding cassette subfamily B member 1
AI: Artificial intelligence
ANN: Artificial neural network
CNN: Convolutional neural network
CTLA4: Cytotoxic T-lymphocyte-associated protein 4
CYP: Cytochrome P450
GA: Genetic algorithm
GA-BPNN: Genetic algorithm back-propagation neural network
GBDT: Gradient boosting decision tree
GLM: Generalized linear model
INR: International normalized ratio
IWPC: International Warfarin Pharmacogenetics Consortium
LASSO: Least absolute shrinkage and selection operator
LightGBM: Light gradient boosting machine
LR: Linear regression
LSTM: Long short-term memory
MAE: Mean absolute error
MARS: Multivariate adaptive regression splines
ML: Machine learning
MLR: Multiple linear regression
NGO-BPANN: Non-greedy optimization-based artificial neural network
PK: Pharmacokinetic
PopPK: Population pharmacokinetic
POR: P450 oxidoreductase
PPAR-α: Peroxisome proliferator-activated receptor alpha
PRISMA: Preferred Reporting Items for Systematic Reviews and Meta-Analyses
PROBAST: Prediction model Risk of Bias Assessment Tool
PW20: Percentage of predictions within ±20% of observed value
R^2^: Coefficient of determination
RF: Random forest
RMSE: Root mean square error
SNP: Single nucleotide polymorphism
SVM: Support vector machine
TAC: Tacrolimus
TRIPOD: Transparent Reporting of a multivariable prediction model for Individual Prognosis Or Diagnosis
UGT2B7: UDP glucuronosyltransferase family 2 member B7
VKORC1: Vitamin K epoxide reductase complex subunit 1
XGBoost: Extreme gradient boosting
