# Supplementary material for: Can artificial intelligence improve precision dosing? a systematic review of narrow therapeutic index drugs
Source: BMC Med Inform Decis Mak. 2026 Aug 17;26:463. doi: 10.1186/s12911-026-03769-7 (PMC13756906; doi:10.1186/s12911-026-03769-7)
Supplement: Supplementary file 2 [file 12911_2026_3769_MOESM3_ESM.docx]

**Supplementary File 2**

**Table S1:** Detailed search strategies used to identify studies evaluating artificial intelligence for precision dosing of narrow therapeutic index drugs across the included electronic databases.

|  | **Search Terms** |
| --- | --- |
| **PubMed** | |
| #1 | ( dose[Title/Abstract] OR dosage [Title/Abstract] OR dosing[Title/Abstract] OR "dose adjustment" [Title/Abstract] OR "dose optimization"[Title/Abstract] OR "dose individualization" [Title/Abstract] OR "personalized dosing"[Title/Abstract] OR "dose prediction"[Title/Abstract] OR "dose calculation" [Title/Abstract]) |
| #2 | ( "artificial intelligence"[Title/Abstract] OR AI[Title/Abstract] OR "machine learning"[Title/Abstract] OR "deep learning"[Title/Abstract]) |
| #3 | (pharmacogenomics [Title/Abstract] OR pgx[Title/Abstract] OR genomic [Title/Abstract] OR genetics [Title/Abstract] OR genotype [Title/Abstract]) |
| #4 | #1 AND #2 AND #3 |
| **Scopus** | |
| #1 | ("drug dos*" OR "dose regim*" OR dosing OR "dose adjustment" OR "dose optimization" OR "dose individualization" OR "personalized dosing" OR "dose prediction" OR "dose calculation" OR ( drug W/3 dos*) ) |
| #2 | ("artificial intelligence" OR AI OR "machine learning" OR "deep learning") |
| #3 | (pharmacogenomics OR pgx OR "genomic" OR "genetics" OR "genotype" ) AND ( "artificial intelligence" OR AI OR "machine learning" OR "deep learning") |
| #4 | #1 AND #2 AND #3 AND (LIMIT-TO (LANGUAGE, "English")) |
| **Cochrane library** | |
| #1 | ("artificial intelligence" OR "machine learning" OR "deep learning" OR AI ) |
| #2 | (dosing OR dosage OR dose OR dosing OR "dose adjustment" OR "dose optimization" OR "dose individualization" OR "personalized dosing" OR "dose prediction" OR "dose calculation") |
| #3 | (pharmacogenomics OR pgx OR genomic OR genetics OR genotype) |
| #4 | #1 AND #2 AND #3 |
| **IEEE Xplore** | |
| #1 | ("artificial intelligence" OR "machine learning" OR "deep learning" OR AI) |
| #2 | (dose OR dosage OR dosing OR "dose adjustment" OR "dose optimization" OR "dose individualization" OR "personalized dosing" OR "dose prediction" OR "dose calculation") |
| #3 | ("pharmacogenomics" OR "pgx" OR "genomic" OR "genetics" OR "genotype") |
| #4 | #1 AND #2 AND #3 |

**Table S2:** Detailed inclusion and exclusion criteria used for study selection according to the PICOS framework

| **PICOS** | **Inclusion Criteria** | **Exclusion Criteria** |
| --- | --- | --- |
| **Population (P)** | Human participants receiving narrow therapeutic index (NTI) drugs (e.g., warfarin, tacrolimus, cyclosporine, valproic acid, oxcarbazepine, or other NTI drugs). | Animal studies, in vitro studies, studies not involving NTI drugs, or studies without human participants. |
| **Intervention (I)** | Artificial intelligence (AI) or machine learning (ML) models developed, validated, or evaluated for individualized drug dosing or prediction of drug dose, concentration, or exposure. | Studies not involving AI/ML-based prediction models. |
| **Comparator (C)** | Conventional dosing methods, pharmacokinetic models, Bayesian approaches, linear/statistical regression models, clinical dosing algorithms, alternative AI/ML models, or studies without a comparator if predictive model performance was reported. | Studies without sufficient evaluation of predictive model performance. |
| **Outcomes (O)** | Model performance measures (e.g., R², MAE, RMSE, PW20, prediction error, calibration, discrimination, or other predictive accuracy metrics related to individualized dosing). | Studies not reporting predictive model performance or clinically relevant prediction outcomes. |
| **Study design (S)** | Original peer-reviewed studies describing the development, validation, or evaluation of AI/ML models for individualized dosing. | Reviews, systematic reviews, meta-analyses, conference abstracts, editorials, letters, protocols, case reports, and studies lacking extractable outcomes. |

**Table S3**: Detailed screening checklist for all records assessed during phase 1 and phase 2 screening, including eligibility and final inclusion or exclusion decisions

| **No** | **Title** | **Population** | **Intervention** | **Comparator** | **Outcome** | **Study type** | **SUM** | **Decision** |
| --- | --- | --- | --- | --- | --- | --- | --- | --- |
| 1 | VPA Metabolism in Neurosurgery | 1 | 1 | 1 | 1 | 1 | 5 | Included |
| 2 | Warfit-learn Evaluation & Evolutionary Optimization | 1 | 1 | 1 | 1 | 1 | 5 | Included |
| 3 | ML for Warfarin in Caribbean Hispanics | 1 | 1 | 1 | 1 | 1 | 5 | Included |
| 4 | LSTM for individualized INR prediction | 1 | 1 | 1 | 1 | 1 | 5 | Included |
| 5 | Warfarin Dosing Prediction in Korean Population | 1 | 1 | 1 | 1 | 1 | 5 | Included |
| 6 | Tacrolimus Levels in Pediatric SOT Recipients | 1 | 1 | 1 | 1 | 1 | 5 | Included |
| 7 | Tacrolimus Dose Prediction in Chinese Kidney Transplant | 1 | 1 | 1 | 1 | 1 | 5 | Included |
| 8 | Optimizing Tacrolimus Dosage using DoseOptimal Framework | 1 | 1 | 1 | 1 | 1 | 5 | Included |
| 9 | MHD Concentration Prediction in Epilepsy Patients | 1 | 1 | 1 | 1 | 1 | 5 | Included |
| 10 | Warfarin Dose Prediction with Stratified Sampling | 1 | 1 | 1 | 1 | 1 | 5 | Included |
| 11 | Warfarin Dose Prediction in Northern Chinese patients | 1 | 1 | 1 | 1 | 1 | 5 | Included |
| 12 | Tacrolimus PK in Pediatric Nephrotic Syndrome | 1 | 1 | 1 | 1 | 1 | 5 | Included |
| 13 | Tacrolimus Dosing with Deep Forest and GA | 1 | 1 | 1 | 1 | 1 | 5 | Included |
| 14 | MLAs for tacrolimus and cyclosporine in renal transplants | 1 | 1 | 1 | 1 | 1 | 5 | Included |
| 15 | Hybrid BP-GA model for heart valve replacement | 1 | 1 | 1 | 1 | 1 | 5 | Included |
| 16 | Heuristic-stacking for warfarin in China | 1 | 1 | 1 | 1 | 1 | 5 | Included |
| 17 | Supervised learning approach for predicting warfarin dosage. | 1 | 1 | 1 | 1 | 0 | 4 | Excluded |
| 18 | Revisiting warfarin dosing using machine learning techniques (Sharabiani). | 1 | 1 | 1 | 1 | 0 | 4 | Excluded |
| 19 | Ensemble of machine learning algorithms using stacked generalization. | 1 | 1 | 1 | 1 | 0 | 4 | Excluded |
| 20 | New personalized algorithm for Chinese heart valve replacement (Tan). | 1 | 1 | 1 | 1 | 0 | 4 | Excluded |
| 21 | Artificial Intelligence in Drug Treatment | 1 | 1 | 1 | 1 | 0 | 4 | Excluded |
| 22 | AI Models for Predicting Drug Efficacy and Toxicity | 1 | 1 | 0 | 0 | 0 | 2 | Excluded |
| 23 | Precision Psychiatry Applications with PGx | 1 | 1 | 0 | 0 | 0 | 2 | Excluded |
| 24 | Differentiating Separate Primary Lung Adenocarcinomas | 1 | 1 | 0 | 0 | 0 | 2 | Excluded |
| 25 | Personalized Brachytherapy: Future Directions | 1 | 1 | 0 | 0 | 0 | 2 | Excluded |
| 26 | Radiomics and deep learning in lung cancer | 1 | 1 | 0 | 0 | 0 | 2 | Excluded |
| 27 | Genomics and the early diagnosis of lung cancer | 1 | 1 | 0 | 0 | 0 | 2 | Excluded |
| 28 | Global perspectives on Myopia: Precision Medicine | 1 | 1 | 0 | 0 | 0 | 2 | Excluded |
| 29 | PGx Beyond Single Common Genetic Variants | 1 | 1 | 0 | 0 | 0 | 2 | Excluded |
| 30 | ANN and bioavailability of immunosuppression drug | 1 | 1 | 0 | 0 | 0 | 2 | Excluded |
| 31 | Role of AI in Musculoskeletal Interventions | 1 | 1 | 0 | 0 | 0 | 2 | Excluded |
| 32 | Normal Tissue Toxicity Prediction: Translation | 1 | 1 | 0 | 0 | 0 | 2 | Excluded |
| 33 | New Drug Development and Clinical Trial Design | 1 | 1 | 0 | 0 | 0 | 2 | Excluded |
| 34 | Pharmaceutical care model in China | 1 | 1 | 0 | 0 | 0 | 2 | Excluded |
| 35 | Predictive Modelling in pharmacokinetics | 1 | 1 | 0 | 0 | 0 | 2 | Excluded |
| 36 | PGx and rare diseases: optimizing drug development | 1 | 1 | 0 | 0 | 0 | 2 | Excluded |
| 37 | Advances in personalized medicine... transplantation | 1 | 1 | 0 | 0 | 0 | 2 | Excluded |
| 38 | Advancements in the treatment of AMD | 1 | 1 | 0 | 0 | 0 | 2 | Excluded |
| 39 | Recent advances in precision medicine: immunosuppression | 1 | 1 | 0 | 0 | 0 | 2 | Excluded |
| 40 | Novel radiotherapeutic strategies: brain metastases | 1 | 1 | 0 | 0 | 0 | 2 | Excluded |
| 41 | Emerging Therapies in ADPKD | 1 | 0 | 0 | 0 | 0 | 1 | Excluded |
| 42 | Integrating Molecular Graph Data... Drug Response | 1 | 1 | 0 | 1 | 1 | 4 | Excluded |
| 43 | Genomic and Imaging Biomarker Analysis in Lung Cancer | 1 | 1 | 0 | 0 | 1 | 3 | Excluded |
| 44 | Impact of PGx Testing on Cost Effectiveness... | 1 | 0 | 1 | 1 | 1 | 4 | Excluded |
| 45 | ML-Based Prediction of Impulse Control Disorders | 1 | 1 | 1 | 1 | 1 | 5 | Excluded |
| 46 | Generalizable multi-vaccine distribution strategy | 0 | 1 | 0 | 0 | 1 | 2 | Excluded |
| 47 | Framework for assessing responses/biomarkers | 1 | 1 | 0 | 1 | 1 | 4 | Excluded |
| 48 | Toxicogenomic data space for understanding EDCs | 1 | 1 | 0 | 1 | 1 | 4 | Excluded |
| 49 | ML-Based Segmentation of Bone Marrow Imaging | 1 | 1 | 0 | 0 | 1 | 3 | Excluded |
| 50 | Glomerulus Classification via an Improved GoogLeNet | 1 | 1 | 0 | 0 | 1 | 3 | Excluded |
| 51 | A cross-disorder dosage sensitivity map | 1 | 1 | 0 | 0 | 1 | 3 | Excluded |
| 52 | Evolution of Personalized Medicine: Big Data | 1 | 1 | 1 | 1 | 0 | 4 | Excluded |
| 53 | Personalised Exercise Training in Adolescents... | 0 | 1 | 1 | 1 | 1 | 4 | Excluded |
| 54 | Validation of a Cell-Free DNA Fragmentome Assay | 1 | 1 | 1 | 0 | 1 | 4 | Excluded |
| 55 | Risk Assessment in Oncology using Big Data | 1 | 1 | 0 | 1 | 0 | 3 | Excluded |
| 56 | Various Cancer Analysis Using Big Data | 1 | 1 | 0 | 1 | 0 | 3 | Excluded |
| 57 | Modern feeding strategies for breeding boars | 0 | 0 | 0 | 0 | 0 | 0 | Excluded |
| 58 | Personalized Perioperative Opioid Strategies in Children. Review | 1 | 0 | 0 | 0 | 0 | 1 | Excluded |
| 59 | Therapeutics and PGx of Brolucizumab... Deep Learning | 1 | 1 | 1 | 0 | 1 | 4 | Excluded |
| 60 | Factors Influencing MPA EHC... XGboost model | 1 | 1 | 1 | 0 | 1 | 4 | Excluded |
| 61 | Effect of Longitudinal Data on Warfarin Dose Predictions | 1 | 1 | 1 | 1 | 0 | 4 | Excluded |
| 62 | Warfarin Dosage using XGBoost-based ML Model | 1 | 1 | 1 | 1 | 0 | 4 | Excluded |
| 63 | Automated warfarin dose prediction for Asian/Caucas. | 1 | 1 | 1 | 1 | 0 | 4 | Excluded |
| 64 | Evaluation of supervised ML algorithms for Warfarin | 1 | 1 | 1 | 1 | 0 | 4 | Excluded |
| 65 | Predicting Range of Initial Warfarin Dose... Pharmacometabolomic | 1 | 1 | 1 | 0 | 1 | 4 | Excluded |
| 66 | ML advances integration of covariates... Valproic acid | 1 | 1 | 1 | 0 | 1 | 4 | Excluded |
| 67 | Advanced ML Approaches to PGx Recommendations | 1 | 0 | 1 | 1 | 1 | 4 | Excluded |
| 68 | Hybrid PD/ML model for chemotherapy neutropenia | 1 | 0 | 1 | 1 | 1 | 4 | Excluded |
| 69 | Improved stacking ensemble learning for warfarin | 1 | 1 | 1 | 1 | 0 | 4 | Excluded |
| 70 | Simulating clinical trials for MIPD: Warfarin | 1 | 1 | 1 | 1 | 0 | 4 | Excluded |
| 71 | Latent batch-constrained deep RL for precision dosing | 1 | 1 | 1 | 1 | 0 | 4 | Excluded |
| 72 | Post-Hoc Interpretable Ensemble for Warfarin | 1 | 1 | 1 | 1 | 0 | 4 | Excluded |
| 73 | iDIS predicts in vivo TB drug interactions | 1 | 0 | 1 | 1 | 1 | 4 | Excluded |
| 74 | Warfit-learn: Warfarin dosing research | 1 | 1 | 1 | 1 | 0 | 4 | Excluded |
| 75 | Algorithmic RNA biomarkers for individual response | 1 | 0 | 1 | 1 | 1 | 4 | Excluded |
| 76 | BP-GA Hybrid for Multi-Herb Optimization | 1 | 0 | 1 | 1 | 1 | 4 | Excluded |
| 77 | Developing ML algorithms to evaluate tacrolimus/cyclosporine in renal transplants | 1 | 1 | 1 | 0 | 1 | 4 | Excluded |
| 78 | Predictive models for fentanyl dose requirement in major breast surgery | 1 | 1 | 1 | 0 | 1 | 4 | Excluded |
| 79 | Quantum Deep RL for Clinical Decision... Radiotherapy | 1 | 1 | 1 | 1 | 1 | 5 | Excluded |
| 80 | Pharmacogenomics drives lenalidomide efficacy... MCL0208 | 1 | 1 | 1 | 0 | 1 | 4 | Excluded |
| 81 | Optimizing glycemic control with AI-guided dietary supplements | 0 | 1 | 1 | 1 | 1 | 4 | Excluded |
| 82 | POTENCY OF SINGLE VS DOUBLE DOSE RITUXIMAB... | 1 | 0 | 1 | 1 | 1 | 4 | Excluded |
| 83 | Safety and immunogenicity of TG4050: personalized vaccine | 1 | 0 | 1 | 1 | 1 | 4 | Excluded |
| 84 | Protocol amendment for ENSEMBLE study: LARC | 1 | 1 | 1 | 0 | 1 | 4 | Excluded |
| 85 | Personalising Antidepressant Treatment: PETRUSHKA Tool | 1 | 1 | 1 | 0 | 1 | 4 | Excluded |
| 86 | Proteogenomic profiling predicts outcomes of EH-CCA | 1 | 1 | 1 | 0 | 1 | 4 | Excluded |
| 87 | HIV incidence after PrEP initiation: risk score | 1 | 1 | 0 | 1 | 1 | 4 | Excluded |
| 88 | Neurobiological Predictors of Response to Neurostimulation | 1 | 1 | 1 | 0 | 1 | 4 | Excluded |
| 89 | RhPro-UK in acute ischemic stroke: PROST-2 | 1 | 0 | 1 | 1 | 1 | 4 | Excluded |
| 90 | Metformin-Lifestyle in antipsychotic users: MELIA trial | 1 | 1 | 1 | 0 | 1 | 4 | Excluded |
| 91 | Thrombolysis with RhPro-UK 4.5-6 h: Phase IIa | 1 | 0 | 1 | 1 | 1 | 4 | Excluded |
| 92 | Evaluation of AI system for estrus detection in sows | 0 | 1 | 1 | 1 | 1 | 4 | Excluded |
| 93 | SYNPRED: prediction of drug combination effects in cancer | 1 | 1 | 0 | 1 | 1 | 4 | Excluded |
| 94 | Supporting Machine Learning Model in the Treatment of Chronic Pain | 1 | 1 | 1 | 0 | 1 | 4 | Excluded |
| 95 | ML Application in Phase I Trial for clinical-biomolecular markers of Toxicity | 1 | 1 | 1 | 0 | 1 | 4 | Excluded |
| 96 | Dietary Mg, CRP and IL-6: The Strong Heart Family Study | 1 | 0 | 1 | 1 | 1 | 4 | Excluded |
| 97 | High-dose melphalan treatment increases mutational burden in MM | 1 | 1 | 1 | 0 | 1 | 4 | Excluded |
| 98 | Predicting Chemotherapy-Induced Peripheral Neuropathy Using Transformer | 1 | 1 | 1 | 0 | 1 | 4 | Excluded |
| 99 | Validating Mathematical Model-Based PGx Dose Prediction | 1 | 0 | 1 | 1 | 1 | 4 | Excluded |
| 100 | Ruxolitinib and exemestane in breast cancer | 1 | 0 | 1 | 1 | 1 | 4 | Excluded |
| 101 | Upfront high-dose consolidation in follicular lymphoma | 1 | 1 | 1 | 0 | 1 | 4 | Excluded |
| 102 | 2020 breakthroughs in secondary prevention: Review | 1 | 0 | 1 | 1 | 0 | 3 | Excluded |
| 103 | CNVoyant: ML framework for copy number variant classification | 1 | 1 | 0 | 0 | 1 | 3 | Excluded |
| 104 | Risk prediction for delayed clearance of high-dose methotrexate... ML | 1 | 1 | 0 | 0 | 1 | 3 | Excluded |
| 105 | ML and Systems Biology to Characterize Dosage-Based Gene Dependencies | 1 | 1 | 0 | 0 | 1 | 3 | Excluded |
| 106 | Dopaminergic Gene Dosage Reveals Distinct Biological Partitions... ASD | 1 | 1 | 0 | 0 | 1 | 3 | Excluded |
| 107 | PET/CT and ML Prediction of pathological complete response in TNBC | 1 | 1 | 0 | 0 | 1 | 3 | Excluded |
| 108 | Age-specific risk prediction models for POI in childhood cancer survivors | 1 | 1 | 0 | 0 | 1 | 3 | Excluded |
| 109 | Non-invasive identification of TKI-resistant NSCLC: AI approach | 1 | 1 | 0 | 0 | 1 | 3 | Excluded |
| 110 | ML to predict high-dose methotrexate-related neutropenia and fever | 1 | 1 | 0 | 0 | 1 | 3 | Excluded |
| 111 | Dose-response modeling in high-throughput cancer drug screenings | 0 | 1 | 0 | 1 | 1 | 3 | Excluded |
| 112 | CREAMMIST: integrative probabilistic database for response prediction | 1 | 1 | 0 | 0 | 1 | 3 | Excluded |
| 113 | CNVoyant: Highly Performant Multi-Classifier for CNVs | 1 | 1 | 0 | 0 | 1 | 3 | Excluded |
| 114 | Variegation of autism related traits across seven neurogenetic disorders | 1 | 1 | 0 | 0 | 1 | 3 | Excluded |
| 115 | Investigation of liquid biopsy analytes... SARS-CoV-2 | 1 | 1 | 0 | 0 | 1 | 3 | Excluded |
| 116 | Genomic Profiling and Drug Screening... Neuroblastoma | 1 | 1 | 0 | 0 | 1 | 3 | Excluded |
| 117 | Community challenge for MoA inference | 1 | 1 | 0 | 0 | 1 | 3 | Excluded |
| 118 | Predicting second cancer with ML models | 1 | 1 | 0 | 0 | 1 | 3 | Excluded |
| 119 | ML models for predicting risks of MACEs | 1 | 1 | 0 | 0 | 1 | 3 | Excluded |
| 120 | Functional assessment of pharmacogenomic variants | 1 | 1 | 0 | 0 | 1 | 3 | Excluded |
| 121 | ML for early prediction of secondary cancer | 1 | 1 | 0 | 0 | 1 | 3 | Excluded |
| 122 | ApoA-I and CAD: Mendelian randomization | 1 | 0 | 1 | 0 | 1 | 3 | Excluded |
| 123 | Personalized outcome prediction in lung cancer screening | 1 | 1 | 0 | 0 | 1 | 3 | Excluded |
| 124 | Discovering gene-brain-behavior link in autism | 1 | 1 | 0 | 0 | 1 | 3 | Excluded |
| 125 | Physical activity, genetics, and back pain risk | 1 | 1 | 0 | 0 | 1 | 3 | Excluded |
| 126 | Integration of Dose Surface Maps and Genetic Data | 1 | 1 | 0 | 0 | 1 | 3 | Excluded |
| 127 | Body composition analysis in NSCLC using AI | 1 | 1 | 0 | 0 | 1 | 3 | Excluded |
| 128 | 61st Annual Conference of Indian Society of Gastroenterology | 1 | 1 | 0 | 0 | 0 | 2 | Excluded |
| 129 | bull fertility-associated antigen (Bulls) | 0 | 0 | 0 | 1 | 1 | 2 | Excluded |
| 130 | phase Ia/Ib study of NPX887: B7-H7 | 1 | 0 | 0 | 1 | 0 | 2 | Excluded |
| 131 | STELLAR-304: zanzalintinib and nivolumab | 1 | 0 | 1 | 0 | 0 | 2 | Excluded |
| 132 | Diagnostic, Therapeutic, and Prognostic Applications of AI in Brain Metastases | 1 | 1 | 0 | 0 | 0 | 2 | Excluded |
| 133 | Natural growth history of SSN: Radiology, genetics, and clinical management | 1 | 1 | 0 | 0 | 0 | 2 | Excluded |
| 134 | Development of ND-AI bivalent vector vaccines in commercial chickens | 0 | 0 | 0 | 1 | 1 | 2 | Excluded |
| 135 | Role of Pharmacogenetics in Adverse Drug Reactions: Update | 1 | 1 | 0 | 0 | 0 | 2 | Excluded |
| 136 | Pharmacogenomics and mutation informatics... isoniazid. Review | 1 | 1 | 0 | 0 | 0 | 2 | Excluded |
| 137 | Smart dosing: revolutionizing uveal melanoma treatment with AI. Review | 1 | 1 | 0 | 0 | 0 | 2 | Excluded |
| 138 | Advancing Precision Medicine: Innovative In Silico Approaches. Review | 1 | 1 | 0 | 0 | 0 | 2 | Excluded |
| 139 | Role of AI and ML in thoracic surgery. Review | 1 | 1 | 0 | 0 | 0 | 2 | Excluded |
| 140 | Lnclocator-imb: Ensemble DL for RNA Subcellular Localization | 0 | 1 | 0 | 0 | 1 | 2 | Excluded |
| 141 | Revolutionizing Utility of Big Data Analytics in Cardiovascular Care. Review | 1 | 1 | 0 | 0 | 0 | 2 | Excluded |
| 142 | Platinum-Based Chemotherapeutics review | 1 | 1 | 0 | 0 | 0 | 2 | Excluded |
| 143 | Machine Learning-Assisted Biosensor response in Yeast | 0 | 1 | 0 | 0 | 1 | 2 | Excluded |
| 144 | Predictive medical approaches to hypertension management | 1 | 1 | 0 | 0 | 0 | 2 | Excluded |
| 145 | Superovulation effect on fertility in dairy heifers | 0 | 0 | 0 | 1 | 1 | 2 | Excluded |
| 146 | Efficacy of H5 vaccines in commercial broilers | 0 | 0 | 0 | 1 | 1 | 2 | Excluded |
| 147 | Molecular determinants of aneuploidy in wild yeast | 0 | 1 | 0 | 0 | 1 | 2 | Excluded |
| 148 | GWAS and Prediction of Salt Tolerance in Alfalfa | 0 | 1 | 0 | 0 | 1 | 2 | Excluded |
| 149 | AMR in Ophthalmology review | 1 | 1 | 0 | 0 | 0 | 2 | Excluded |
| 150 | Personalizing Antidepressant Therapy review | 1 | 1 | 0 | 0 | 0 | 2 | Excluded |
| 151 | Cardioprotective effects of exercise in APOE mice | 0 | 1 | 0 | 0 | 1 | 2 | Excluded |
| 152 | Effect of Spirulina on immune response in broiler chickens | 0 | 0 | 0 | 1 | 1 | 2 | Excluded |
| 153 | Chronic β-Blockade review | 1 | 1 | 0 | 0 | 0 | 2 | Excluded |
| 154 | GenoSiS: Genotype Similarity Search Architecture | 1 | 1 | 0 | 0 | 0 | 2 | Excluded |
| 155 | Gdaphen: R pipeline for predictor variables | 1 | 1 | 0 | 0 | 0 | 2 | Excluded |
| 156 | IMPACT OF DUAL-ACTIVE INGREDIENT LLINs (Mosquitoes) | 0 | 0 | 0 | 0 | 1 | 1 | Excluded |
| 157 | Clinical Pharmacology in Drug Development for Rare Diseases... Review | 1 | 0 | 0 | 0 | 0 | 1 | Excluded |
| 158 | Next-Generation Anticoagulants: Precision Strategies. Review | 1 | 0 | 0 | 0 | 0 | 1 | Excluded |
| 159 | Recent advances in lung cancer research: unravelling the future | 1 | 0 | 0 | 0 | 0 | 1 | Excluded |
| 160 | Systematic Review of Pharmacogenomics CDSS Used in Clinical Practice | 1 | 0 | 0 | 0 | 0 | 1 | Excluded |
| 161 | Research landscape of precision medicine in cardiology. Review | 1 | 0 | 0 | 0 | 0 | 1 | Excluded |
| 162 | Adjuvant systemic therapy decisions in older breast cancer patients. Review | 1 | 0 | 0 | 0 | 0 | 1 | Excluded |
| 163 | Lung cancer screening in India: Preparing for the future. Review | 1 | 0 | 0 | 0 | 0 | 1 | Excluded |
| 164 | Effects of Targeted Radionuclide Therapy Beyond Ablative Dose. Review | 1 | 0 | 0 | 0 | 0 | 1 | Excluded |
| 165 | Resveratrol as antioxidant for boar semen review | 0 | 0 | 0 | 0 | 0 | 0 | Excluded |
| 166 | Systematic review and meta-analysis of AI accuracy in warfarin dose prediction... | 1 | 1 | 1 | 1 | 0 | 4 | Excluded |
| 167 | Implementation of model-informed precision dosing for tamoxifen therapy... | 1 | 0 | 1 | 1 | 1 | 4 | Excluded |
| 168 | New potential therapeutic approach for ALS (Case Report) | 1 | 1 | 1 | 1 | 0 | 4 | Excluded |
| 169 | XGraphCDS: Predicting drug sensitivity... | 1 | 1 | 0 | 1 | 1 | 4 | Excluded |
| 170 | Factors for adrenal crisis in chronic AI | 1 | 0 | 1 | 1 | 1 | 4 | Excluded |
| 171 | Adrenocortical function in SLSMDs | 1 | 0 | 1 | 1 | 1 | 4 | Excluded |
| 172 | Transcriptome analyses of gene therapy for XLMTM | 1 | 1 | 0 | 1 | 1 | 4 | Excluded |
| 173 | Voriconazole therapy in liver dysfunction... | 1 | 0 | 1 | 1 | 1 | 4 | Excluded |
| 174 | AIs and contralateral breast cancer in BRCA | 1 | 0 | 1 | 1 | 1 | 4 | Excluded |
| 175 | Probiotics in obesity: Microbiota functions | 1 | 0 | 1 | 1 | 1 | 4 | Excluded |
| 176 | ML vs Logistic Regression for Methotrexate | 1 | 1 | 0 | 1 | 1 | 4 | Excluded |
| 177 | Pharmacogenomics of AIs in Breast Cancer | 1 | 0 | 1 | 1 | 1 | 4 | Excluded |
| 178 | Interaction between FADS1 and vegetable oils | 1 | 0 | 1 | 1 | 1 | 4 | Excluded |
| 179 | Effects of Recombinant Human LCAT on Lipoprotein | 1 | 0 | 1 | 1 | 1 | 4 | Excluded |
| 180 | Psilocybin therapy for TRD: prediction by NLP | 1 | 1 | 1 | 0 | 1 | 4 | Excluded |
| 181 | Identifying Gut Microbiome Features... Prebiotic | 1 | 1 | 1 | 0 | 1 | 4 | Excluded |
| 182 | Safety results of PADA-1: AI + palbociclib | 1 | 0 | 1 | 1 | 1 | 4 | Excluded |
| 183 | Predictive endocrine resistance index (PERCI) | 1 | 0 | 1 | 1 | 1 | 4 | Excluded |
| 184 | PK, USABILITY AND SAFETY OF TOCILIZUMAB BIOSIMILAR | 1 | 0 | 1 | 1 | 1 | 4 | Excluded |
| 185 | Immunometabolic Effects of Dietary Fibers | 1 | 1 | 1 | 0 | 1 | 4 | Excluded |
| 186 | Efficacy and safety of ongericimab: hypercholesterolemia | 1 | 0 | 1 | 1 | 1 | 4 | Excluded |
| 187 | Postprandial remodeling of HDL | 1 | 0 | 1 | 1 | 1 | 4 | Excluded |
| 188 | COLUMBUS 7-year update: Melanoma | 1 | 0 | 1 | 1 | 1 | 4 | Excluded |
| 189 | Transcriptomic Predictors of Survival: PEARL Trial | 1 | 1 | 1 | 0 | 1 | 4 | Excluded |
| 190 | Lasofoxifene Versus Fulvestrant: ELAINE 1 | 1 | 0 | 1 | 1 | 1 | 4 | Excluded |
| 191 | WSG-ADAPT HR+/HER2- chemotherapy trial | 1 | 0 | 1 | 1 | 1 | 4 | Excluded |
| 192 | Adjuvant hormone therapy: UNIRAD trial | 1 | 0 | 1 | 1 | 1 | 4 | Excluded |
| 193 | endocrine adjuvant therapy intake timing | 1 | 0 | 1 | 1 | 1 | 4 | Excluded |
| 194 | EFFECT OF OLEZARSEN ON APOC-III | 1 | 0 | 1 | 1 | 1 | 4 | Excluded |
| 195 | LEFAMULIN EFFICACY AND SAFETY: LEAP 1 and 2 | 1 | 0 | 1 | 1 | 1 | 4 | Excluded |
| 196 | Standard of care with or without SABR: Ovarian Cancer | 1 | 1 | 1 | 0 | 1 | 4 | Excluded |
| 197 | Tailored dose-dense chemotherapy: PANTHER trial | 1 | 0 | 1 | 1 | 1 | 4 | Excluded |
| 198 | Pembrolizumab vs placebo... resected melanoma | 1 | 0 | 1 | 1 | 1 | 4 | Excluded |
| 199 | Phase Ib single-arm trial of bintrafusp alfa (BA)... | 1 | 0 | 1 | 1 | 1 | 4 | Excluded |
| 200 | Nivolumab plus relatlimab vs nivolumab... | 1 | 0 | 1 | 1 | 1 | 4 | Excluded |
| 201 | Safety and efficacy of tebentafusp (tebe) in mUM | 1 | 0 | 1 | 1 | 1 | 4 | Excluded |
| 202 | Integrative and deep learning-based prediction of therapy response in ovarian cancer | 1 | 1 | 0 | 0 | 1 | 3 | Excluded |
| 203 | Integrating AI-driven digital pathology and genomics... for drug response | 1 | 1 | 0 | 0 | 1 | 3 | Excluded |
| 204 | Immunophenotype-guided interpretable radiomics model for anti-PD-1 response... | 1 | 1 | 0 | 0 | 1 | 3 | Excluded |
| 205 | Epigenetic Heritability of Cell Plasticity Drives Cancer Drug Resistance... | 1 | 1 | 0 | 0 | 1 | 3 | Excluded |
| 206 | Circadian rhythm disruption by PARP inhibitors correlates with toxicity... | 1 | 1 | 0 | 0 | 1 | 3 | Excluded |
| 207 | Effect of Cumulative Exposure on the Efficacy of Paroxetine: PopPK/PD and ML | 1 | 1 | 0 | 0 | 1 | 3 | Excluded |
| 208 | CAN-Scan: A multi-omic phenotype-driven precision oncology platform... | 1 | 1 | 0 | 0 | 1 | 3 | Excluded |
| 209 | A deep-learning model for predicting TKI response from histology in GIST | 1 | 1 | 0 | 0 | 1 | 3 | Excluded |
| 210 | DD-PRiSM: a deep learning framework for prediction of synergistic drug combinations | 1 | 1 | 0 | 0 | 1 | 3 | Excluded |
| 211 | A Predictive Approach For Assessing Medication Responsiveness in Breast Cancer | 1 | 1 | 0 | 0 | 1 | 3 | Excluded |
| 212 | The role of gut microbiota... in modulating the efficacy of SSRIs for MDD | 1 | 1 | 0 | 0 | 1 | 3 | Excluded |
| 213 | Single-cell transcriptomes identify therapies for co-inhibition of cancer clones | 1 | 1 | 0 | 0 | 1 | 3 | Excluded |
| 214 | A random survival forest-based pathomics signature... in ES-SCLC patients | 1 | 1 | 0 | 0 | 1 | 3 | Excluded |
| 215 | AI-powered omics-based drug pair discovery for pyroptosis therapy in TNBC | 1 | 1 | 0 | 0 | 1 | 3 | Excluded |
| 216 | Metabolic Response to Small Molecule Therapy in CRC Tracked with RS | 1 | 1 | 0 | 0 | 1 | 3 | Excluded |
| 217 | Impact of SLC22A1 variants... on metformin plasmatic concentration in T2DM | 1 | 1 | 0 | 0 | 1 | 3 | Excluded |
| 218 | Single-Cell Transcriptomic... Reveal Hub Genes for CD Therapy Response | 1 | 1 | 0 | 0 | 1 | 3 | Excluded |
| 219 | Impact of SLC22A1 variants... on metformin in T2DM | 1 | 1 | 0 | 0 | 1 | 3 | Excluded |
| 220 | Single-Cell Transcriptomic... Anti-TNFα Response | 1 | 1 | 0 | 0 | 1 | 3 | Excluded |
| 221 | B7-H4–Targeting Antibody–Drug Conjugate... | 1 | 0 | 0 | 1 | 1 | 3 | Excluded |
| 222 | ML models for therapeutic targets in T2DM in rats | 0 | 1 | 0 | 1 | 1 | 3 | Excluded |
| 223 | Nebivolol and Donepezil in ALS | 0 | 1 | 0 | 1 | 1 | 3 | Excluded |
| 224 | Novel TF Machine Learning Model... in TNBC | 1 | 1 | 0 | 0 | 1 | 3 | Excluded |
| 225 | Detection of EGFR mutation by radiomic features | 1 | 1 | 0 | 0 | 1 | 3 | Excluded |
| 226 | PHAROS Study: Encorafenib Plus Binimetinib | 1 | 0 | 0 | 1 | 1 | 3 | Excluded |
| 227 | Multi-omic modeling of antidepressant response | 1 | 1 | 0 | 0 | 1 | 3 | Excluded |
| 228 | Feature selection/ML for CYP2D6 methylation | 1 | 1 | 0 | 0 | 1 | 3 | Excluded |
| 229 | Lipidomic profiling of thiopurine leukopenia | 1 | 1 | 0 | 0 | 1 | 3 | Excluded |
| 230 | ML for prediction of afatinib treatment outcome | 1 | 1 | 0 | 0 | 1 | 3 | Excluded |
| 231 | MLP phenomics... for monitoring drug-response | 1 | 1 | 0 | 0 | 1 | 3 | Excluded |
| 232 | Human Trypanosoma cruzi chronic infection... | 1 | 0 | 0 | 1 | 1 | 3 | Excluded |
| 233 | ML covariate selection for bevacizumab | 1 | 1 | 0 | 0 | 1 | 3 | Excluded |
| 234 | Growth-rate model predicts in vivo tumor response | 1 | 0 | 0 | 1 | 1 | 3 | Excluded |
| 235 | Prognostic Stratification of MM: IAC-50 Model | 1 | 1 | 0 | 0 | 1 | 3 | Excluded |
| 236 | ADME gene polymorphisms on pemetrexed PK | 1 | 0 | 0 | 1 | 1 | 3 | Excluded |
| 237 | ML for prediction of vincristine neuropathy | 1 | 1 | 0 | 0 | 1 | 3 | Excluded |
| 238 | ML Models for Asparaginase-associated Pancreatitis | 1 | 1 | 0 | 0 | 1 | 3 | Excluded |
| 239 | Ingenol promotes thrombopoiesis... | 0 | 1 | 0 | 1 | 1 | 3 | Excluded |
| 240 | Vandetanib plus Everolimus for DIPG | 1 | 0 | 0 | 1 | 1 | 3 | Excluded |
| 241 | TGI and OS modelling in mCRC | 1 | 1 | 0 | 0 | 1 | 3 | Excluded |
| 242 | Sofosbuvir-based regimens for HCV in AN/AI | 1 | 0 | 0 | 1 | 1 | 3 | Excluded |
| 243 | Ki67 as response biomarker to tamoxifen | 1 | 1 | 0 | 0 | 1 | 3 | Excluded |
| 244 | Data-driven analysis of JAK2V617F kinetics | 1 | 1 | 0 | 0 | 1 | 3 | Excluded |
| 245 | Computational analysis... identifies drug targets | 1 | 1 | 0 | 0 | 1 | 3 | Excluded |
| 246 | DLEPS identifies drug candidates... | 0 | 1 | 0 | 1 | 1 | 3 | Excluded |
| 247 | MDD Prediction of Ketamine Response | 1 | 1 | 0 | 0 | 1 | 3 | Excluded |
| 248 | Modeling AD Trajectories with ML | 1 | 1 | 0 | 0 | 1 | 3 | Excluded |
| 249 | Bacterial persisters in long-term infection | 1 | 1 | 0 | 0 | 1 | 3 | Excluded |
| 250 | MIX-Seq: Response profiling across samples | 1 | 1 | 0 | 0 | 1 | 3 | Excluded |
| 251 | ML reveals EPTB drug resistance patterns | 1 | 1 | 0 | 0 | 1 | 3 | Excluded |
| 252 | Radiomics Response Signature in mCRC | 1 | 1 | 0 | 0 | 1 | 3 | Excluded |
| 253 | GWAS of steady-state letrozole concentration | 1 | 0 | 0 | 1 | 1 | 3 | Excluded |
| 254 | AI-powered HER2 Analysis in TRIUMPH Trial | 1 | 1 | 0 | 0 | 1 | 3 | Excluded |
| 255 | Lower WNT16 expression in patients with AIS | 1 | 0 | 1 | 0 | 1 | 3 | Excluded |
| 256 | Molecular determinants of response to PD-L1 blockade | 1 | 1 | 0 | 0 | 1 | 3 | Excluded |
| 257 | Pathological Response Prediction... Esophageal Carcinoma | 1 | 1 | 0 | 0 | 1 | 3 | Excluded |
| 258 | Validation of AI-based solution for breast cancer risk | 1 | 1 | 0 | 0 | 1 | 3 | Excluded |
| 259 | Contrast Enhancement Mammography vs MRI: Con-TRUST | 1 | 1 | 0 | 0 | 1 | 3 | Excluded |
| 260 | CMV viral load kinetics as surrogate endpoints | 1 | 1 | 0 | 0 | 1 | 3 | Excluded |
| 261 | Tenecteplase Treatment in Ischemic Stroke Registry | 1 | 0 | 0 | 1 | 1 | 3 | Excluded |
| 262 | AMEERA-1: Amcenestrant with palbociclib | 1 | 0 | 0 | 1 | 1 | 3 | Excluded |
| 263 | Intranasal oxytocin impacts trauma memories | 0 | 1 | 1 | 0 | 1 | 3 | Excluded |
| 264 | Psilocybin rTMS for Treatment Resistant Depression | 1 | 0 | 1 | 0 | 1 | 3 | Excluded |
| 265 | Experience pooling control arms... phase I platform trial | 1 | 0 | 1 | 0 | 1 | 3 | Excluded |
| 266 | SunRISe-1: phase IIb study of TAR-200... | 1 | 0 | 1 | 0 | 1 | 3 | Excluded |
| 267 | ARIADNE: randomized trial of trastuzumab deruxtecan... | 1 | 0 | 1 | 0 | 1 | 3 | Excluded |
| 268 | Precision engineering of biological function | 0 | 1 | 0 | 1 | 1 | 3 | Excluded |
| 269 | The Role of AI in Personalized Radiation Therapy and Precision Oncology... | 1 | 1 | 0 | 0 | 0 | 2 | Excluded |
| 270 | Personalized Medicine Recommendation Through Genomics AI | 1 | 1 | 0 | 0 | 0 | 2 | Excluded |
| 271 | Factors affecting voriconazole pharmacokinetic variability in critically ill patients... | 1 | 1 | 0 | 0 | 0 | 2 | Excluded |
| 272 | Dynamic transcriptional immune landscape in response to NK-cell therapy... | 1 | 0 | 0 | 0 | 1 | 2 | Excluded |
| 273 | AI coupled to pharmacometrics modelling to tailor malaria and TB treatment... | 1 | 1 | 0 | 0 | 0 | 2 | Excluded |
| 274 | Surgical and radiological outcomes of giant cell tumor of the bone... | 1 | 0 | 0 | 0 | 1 | 2 | Excluded |
| 275 | Plasmonic Fiber Optic Sensing Platform for PK Monitoring of Platinum... | 1 | 0 | 0 | 0 | 1 | 2 | Excluded |
| 276 | Impact of the tumor immune contexture in MSS metastatic colorectal cancer... | 1 | 0 | 0 | 0 | 1 | 2 | Excluded |
| 277 | Machine learning in pharmacogenomics: From target identification to clinical trials | 1 | 1 | 0 | 0 | 0 | 2 | Excluded |
| 278 | Unraveling the mechanisms of Shaoyang Shenggu decoction in treating KOA... | 0 | 1 | 0 | 0 | 1 | 2 | Excluded |
| 279 | Personalized precision integrating AI for tailored treatment in nuclear medicine | 1 | 1 | 0 | 0 | 0 | 2 | Excluded |
| 280 | 2025 White Paper on Recent Issues in Bioanalysis... | 1 | 1 | 0 | 0 | 0 | 2 | Excluded |
| 281 | Challenges in the therapeutic drug monitoring of immunosuppressants | 1 | 1 | 0 | 0 | 0 | 2 | Excluded |
| 282 | AI-Driven Radiotherapy Solutions for Rare and Complex Cancers... | 1 | 1 | 0 | 0 | 0 | 2 | Excluded |
| 283 | Computational techniques for enhancing PK/PD modeling and ADMET prediction | 1 | 1 | 0 | 0 | 0 | 2 | Excluded |
| 284 | Integrative Personalized Oncology: Leveraging Multi-Source Data... | 1 | 1 | 0 | 0 | 0 | 2 | Excluded |
| 285 | Causal Inference for Continuous Multiple Time Point Interventions | 1 | 0 | 0 | 0 | 1 | 2 | Excluded |
| 286 | Adjuvant T-DM1 vs Paclitaxel Plus Trastuzumab... 5-Year Results from ATEMPT | 1 | 0 | 0 | 0 | 1 | 2 | Excluded |
| 287 | Transforming healthcare with patient-centric and AI-powered personalized medicine | 1 | 1 | 0 | 0 | 0 | 2 | Excluded |
| 288 | Unlocking the potential of LLMs in medication review and reconciliation... | 1 | 1 | 0 | 0 | 0 | 2 | Excluded |
| 289 | AI-driven drug discovery and development | 1 | 1 | 0 | 0 | 0 | 2 | Excluded |
| 290 | Transforming healthcare... AI-powered personalized medicine | 1 | 1 | 0 | 0 | 0 | 2 | Excluded |
| 291 | Unlocking the potential of LLMs in medication review... | 1 | 1 | 0 | 0 | 0 | 2 | Excluded |
| 292 | AI-driven drug discovery and development | 1 | 1 | 0 | 0 | 0 | 2 | Excluded |
| 293 | AI Opportunities to Guide Precision Dosing Strategies | 1 | 1 | 0 | 0 | 0 | 2 | Excluded |
| 294 | Diosgenin Inhibits Gastric Cancer Progression... | 0 | 1 | 0 | 0 | 1 | 2 | Excluded |
| 295 | Holistic approach to ADRs... Review | 1 | 1 | 0 | 0 | 0 | 2 | Excluded |
| 296 | AZD8205 ADC, Alone and in Combination | 0 | 0 | 0 | 1 | 1 | 2 | Excluded |
| 297 | AI and personalized medicine... Review | 1 | 1 | 0 | 0 | 0 | 2 | Excluded |
| 298 | Pharmacogenomics in drug metabolism... Review | 1 | 1 | 0 | 0 | 0 | 2 | Excluded |
| 299 | DIO3OS induces AI resistance in breast cancer | 1 | 0 | 0 | 0 | 1 | 2 | Excluded |
| 300 | Predicting HIV drug resistance... sequences | 0 | 1 | 0 | 0 | 1 | 2 | Excluded |
| 301 | ABCB1 SNPs Predictors of PIPN | 1 | 0 | 0 | 0 | 1 | 2 | Excluded |
| 302 | ZFTG improve RA symptoms in rats | 0 | 0 | 0 | 1 | 1 | 2 | Excluded |
| 303 | Biomarkers of Treatment Response in HD | 0 | 0 | 0 | 1 | 1 | 2 | Excluded |
| 304 | YSTB inhibits B cells in CIA rats | 0 | 0 | 0 | 1 | 1 | 2 | Excluded |
| 305 | SkipGuide: ML for therapeutic exon skipping | 0 | 1 | 0 | 0 | 1 | 2 | Excluded |
| 306 | Decreasing eCG dose in heifers | 0 | 0 | 0 | 1 | 1 | 2 | Excluded |
| 307 | MRI Features under Deep Learning... Propofol Anesthesia | 1 | 0 | 0 | 0 | 1 | 2 | Excluded |
| 308 | Phase I/II study of rinatabart sesutecan (Rina-S)... | 1 | 0 | 0 | 0 | 1 | 2 | Excluded |
| 309 | Evaluate Efficacy, Safety... IV Cipargamin in Malaria | 1 | 0 | 0 | 0 | 1 | 2 | Excluded |
| 310 | Con-RxPONDER: was it all OFS? | 1 | 0 | 1 | 0 | 0 | 2 | Excluded |
| 311 | Low Dose CT Denoising By Concentrating Noise | 0 | 1 | 0 | 0 | 1 | 2 | Excluded |
| 312 | Advancements in 275 nm UV-LED Technology | 0 | 1 | 0 | 0 | 1 | 2 | Excluded |
| 313 | lncLocator-imb: Subcellular Localization | 0 | 1 | 0 | 0 | 1 | 2 | Excluded |
| 314 | ML and Characterize Human Norovirus Susceptibility | 0 | 1 | 0 | 0 | 1 | 2 | Excluded |
| 315 | GEA study: HDL-C/ApoA-I and atherosclerosis | 1 | 0 | 0 | 0 | 1 | 2 | Excluded |
| 316 | A machine learning-based assessment strategy for Artemisia argyi Folium... | 0 | 1 | 0 | 0 | 0 | 1 | Excluded |
| 317 | Chinese expert consensus on precision diagnosis and treatment of refractory tumors | 1 | 0 | 0 | 0 | 0 | 1 | Excluded |
| 318 | Exploration of oxidative stress-mediated genetic toxicology modes of action... | 0 | 1 | 0 | 0 | 0 | 1 | Excluded |
| 319 | Cold receptor TRPM8 as a target for migraine-associated pain... | 0 | 1 | 0 | 0 | 0 | 1 | Excluded |
| 320 | 3,6′-Disinapoyl sucrose modulates GALE-mediated metabolic reprogramming... | 0 | 1 | 0 | 0 | 0 | 1 | Excluded |
| 321 | Dendrobin A inhibits gastric cancer: Mechanistic insights... | 0 | 1 | 0 | 0 | 0 | 1 | Excluded |
| 322 | Pitavastatin overcomes multi-drug resistance in CRC and NSCLC... | 0 | 1 | 0 | 0 | 0 | 1 | Excluded |
| 323 | Deriving safe limits for N-nitroso-bisoprolol by ecNGS and BMD analysis... | 0 | 1 | 0 | 0 | 0 | 1 | Excluded |
| 324 | Jiao-Ai Decoction and active ingredients promoted angiogenesis... | 0 | 1 | 0 | 0 | 0 | 1 | Excluded |
| 325 | Angelman syndrome patient-derived neuron screen leads to rugonersen... | 0 | 1 | 0 | 0 | 0 | 1 | Excluded |
| 326 | Shen Qi Wan regulates OPN/CD44/PI3K pathway to improve COPD... | 0 | 1 | 0 | 0 | 0 | 1 | Excluded |
| 327 | Huanglian Jiedu Decoction enhances the stability of atherosclerotic plaques... | 0 | 1 | 0 | 0 | 0 | 1 | Excluded |
| 328 | The joint toxicity effect of glyphosate and cadmium on C. elegans | 0 | 1 | 0 | 0 | 0 | 1 | Excluded |
| 329 | Updates in Systemic Treatment of Hormone Receptor-Positive Breast Cancer | 1 | 0 | 0 | 0 | 0 | 1 | Excluded |
| 330 | Adaptive Bayesian information borrowing methods for subgroup-specific doses | 1 | 0 | 0 | 0 | 0 | 1 | Excluded |
| 331 | Joint toxicity of glyphosate and cadmium... | 0 | 1 | 0 | 0 | 0 | 1 | Excluded |
| 332 | Updates in Systemic Treatment... Breast Cancer | 1 | 0 | 0 | 0 | 0 | 1 | Excluded |
| 333 | Adaptive Bayesian methods... subgroup-specific doses | 1 | 0 | 0 | 0 | 0 | 1 | Excluded |
| 334 | Cang-ai volatile oil... psoriatic skin lesions | 0 | 1 | 0 | 0 | 0 | 1 | Excluded |
| 335 | Barriers and Unequal Access... Canada | 1 | 0 | 0 | 0 | 0 | 1 | Excluded |
| 336 | TRIM56 protects against NAFLD... | 0 | 1 | 0 | 0 | 0 | 1 | Excluded |
| 337 | Safety and efficacy evaluation of halicin... | 0 | 1 | 0 | 0 | 0 | 1 | Excluded |
| 338 | Enzyme-mediated depletion... MTAP-deficient tumors | 0 | 0 | 0 | 0 | 1 | 1 | Excluded |
| 339 | Biofilm formation of Lactobacillus LIP-1 | 0 | 0 | 0 | 0 | 1 | 1 | Excluded |
| 340 | Effects of FGF21, soluble TGFBR2... on metabolic dysfunction in mice | 0 | 0 | 0 | 0 | 0 | 0 | Excluded |
| 341 | Vitamin D supplementation ameliorates anemia of inflammation... | 0 | 0 | 0 | 0 | 0 | 0 | Excluded |
| 342 | A polytherapy approach demonstrates efficacy for SOD1 associated ALS | 0 | 0 | 0 | 0 | 0 | 0 | Excluded |
| 343 | Integrating multiscale mathematical modeling... on drug resistance in cancer | 0 | 0 | 0 | 0 | 0 | 0 | Excluded |
| 344 | WORLDSymposium™ 2025 Program | 0 | 0 | 0 | 0 | 0 | 0 | Excluded |
| 345 | Protective effect of lappaconitine on adjuvant-induced arthritis in rats | 0 | 0 | 0 | 0 | 0 | 0 | Excluded |
| 346 | Evaluation of the effect of sustained-release progesterone injection in cows | 0 | 0 | 0 | 0 | 0 | 0 | Excluded |
| 347 | Rocaglamide regulates iron homeostasis by suppressing hepcidin expression | 0 | 0 | 0 | 0 | 0 | 0 | Excluded |
| 348 | Effect of sustained-release progesterone... in cows | 0 | 0 | 0 | 0 | 0 | 0 | Excluded |
| 349 | Rocaglamide regulates iron homeostasis... | 0 | 0 | 0 | 0 | 0 | 0 | Excluded |
| 350 | Emergency of SARS-CoV-2: Update | 0 | 0 | 0 | 0 | 0 | 0 | Excluded |
